# DNA methylation differences in monozygotic twins with Van der Woude syndrome

**DOI:** 10.3389/fdmed.2023.1120948

**Published:** 2023-02-17

**Authors:** A. L. Petrin, E. Zeng, M. A. Thomas, D. Moretti-Ferreira, M. L. Marazita, X. J. Xie, J. C. Murray, L. M. Moreno-Uribe

**Affiliations:** ^1^ College of Dentistry and Dental Clinics, University of Iowa, Iowa, IA, United States; ^2^ Departments of Medical Genetics and Pediatrics, Cumming School of Medicine, University of Calgary, Calgary, AB, Canada; ^3^ Department of Chemical and Biological Sciences, Institute of Biosciences, São Paulo State University (UNESP), Botucatu, SP, Brazil; ^4^ Center for Craniofacial and Dental Genetics, University of Pittsburgh, Pittsburgh, PA, United States; ^5^ Carver College of Medicine, University of Iowa, Iowa, IA, United States

**Keywords:** Van der Woude syndrome, DNA methylation, monozygotic twins, phenotypic discordance, cleft lip, lip pits, cleft palate, epigenetics (DNA methylation)

## Abstract

**Introduction:**

Van der Woude syndrome (VWS) is an autosomal dominant disorder responsible for 2% of all syndromic orofacial clefts (OFCs), with *IRF6* being the primary causal gene (70%). Cases may present with lip pits and either cleft lip, cleft lip with cleft palate, or cleft palate, with marked phenotypic discordance even among individuals carrying the same mutation. This suggests that genetic or epigenetic modifiers may play additional roles in the syndrome's etiology and variability in expression. We report the first DNA methylation profiling of two pairs of monozygotic twins with VWS. Our goal is to explore epigenetic contributions to VWS etiology and variable phenotypic expressivity by comparing DNAm profiles in both twin pairs. While the mutations that cause VWS in these twins are known, the additional mechanism behind their phenotypic risk and variability in expression remains unclear.

**Methods:**

We generated whole genome DNAm data for both twin pairs. Differentially methylated positions (DMPs) were selected based on: (1) a coefficient of variation in DNAm levels in unaffected individuals <20%, and (2) intra-twin pair absolute difference in DNAm levels >5% (Δ*β* > |0.05|). We then divided the DMPs into two subgroups for each twin pair for further analysis: (1) higher methylation levels in twin A (Twin A > Twin B); and (2) higher methylation levels in twin B (Twin B >Twin A).

**Results and Discussion:**

Gene ontology analysis revealed a list of enriched genes that showed significant differential DNAm, including clef-associated genes. Among the cleft-associated genes, *TP63* was the most significant hit (p = 7.82E-12). Both twin pairs presented differential DNAm levels in CpG sites in/near *TP63* (Twin 1A > Twin 1B and Twin 2A < Twin 2B). The genes *TP63* and *IRF6* function in a biological regulatory loop to coordinate epithelial proliferation and differentiation in a process that is critical for palatal fusion. The effects of the causal mutations in *IRF6* can be further impacted by epigenetic dysregulation of *IRF6* itself or genes in its pathway. Our data show evidence that changes in DNAm are a plausible mechanism that can lead to markedly distinct phenotypes, even among individuals carrying the same mutation.

## Introduction

While most of the cases of cleft lip and palate are nonsyndromic (1 in 700–2,500 births), orofacial clefts have currently been associated with over 460 syndromes with known molecular basis [OMIM (https://www.omim.org)]. Van der Woude syndrome (VWS) is among the most common syndromic forms of clefts, affecting 1/35,000 individuals (1). It is an autosomal dominant condition that accounts for 2% of all cases with orofacial clefts (2, 3). About 44% of the cases with VWS display lip pits as their sole phenotype (1, 4, 5), but cases with VWS can also show cleft lip (CL), cleft lip and cleft palate (CLP), or cleft palate (CP), all with or without lip pits demonstrating the marked variable expression. Mutations in the gene *IRF6* account for 70% of VWS cases, while mutations in the gene *GRHL3* account for another 5%. However, in approximately 25% of VWS cases, the causal mutation and gene remain unknown.

Mixed clefting types are a common observation in different relatives with VWS within the same family (6–9), further elucidating the variable phenotype observed even among cases carrying the same mutations. Other VWS features include greater prevalence of midfacial hypoplasia and hypodontia (10–12). Moreover, it has been shown that patients with VWS are more likely to have wound complications following cleft repair, including fistulae recurrence (13), and are more likely to require pharyngeal flap surgery (10–12).

*IRF6* is a transcription factor with a highly conserved helix-turn-helix DNA-binding domain and a less conserved SMIR/IAD protein-binding domain (14). *IRF6* is the only member of the IRF gene family involved in craniofacial development, and the mutations that cause VWS are non-randomly distributed, with most occurring in the DNA-binding domain (exons 3 and 4) and the protein-binding domain (exons7–9) (15). Since the discovery of *IRF6* as the first causal gene for VWS (8), more than 300 mutations have been identified in cases with VWS and PPS (15–18).

Although most of the causal mutations are classified as missense mutations, nonsense and frameshift mutations have also been reported. The VWS mutations characterized so far exert their effect on the phenotype *via* haploinsufficiency or a dominant negative effect.

Animal studies have shown that disruption of *IRF6* in mice leads to cleft lip and/or palate (CL/P) in addition to oral epithelial adhesions, poor epithelial barrier functions, and improper skin stratification, which suggests that oral epithelium plays an important role in directing palate development (19, 20). In addition, molecular and histologic analyses showed that *IRF6*-mutated mouse embryos lack periderm cells at the sites of oral adhesions (21), which leads to abnormal epithelial adhesions between the palatal shelves and the lingual, mandibular, and maxillary surfaces, preventing the proper elevation and fusion of the palatal shelves. Besides the abnormal adhesions, the medial edge epithelium (MEE) located at the medial edge of the palatal shelves failed to dissolve for proper palatal fusion also leading to a cleft palate. Similar abnormal bilateral adhesions leading to a cleft palate were observed in mice that were heterozygous for a mutated allele of *GRHL3,* the second VWS locus (22)*.* Thus, both *IRF6* and *GRHL3* are essential to develop a normal oral periderm, which is necessary for palatogenesis (22).

While various established mutations in *IRF6* account for the presence of the syndrome, they do not explain the variability and different levels of phenotypic severity. Since the first publication of *IRF6* being the causal gene, authors have discussed the possible causes of the phenotypic variability observed even among individuals with the same mutation. Studies have suggested the action of stochastic factors or modifier genes on IRF6 function, but epigenetic factors that could play a role remain unexplored. Another important observation is that the mixed clefting phenotype that is common to VWS families is rare in nonsyndromic cleft families. However, this phenotypic variability is also observed in *TP63* (23, 24).

Despite decades of intriguing observations of phenotypic discordance, the first etiological genetic variant for VWS was only discovered in 2002 when Kondo et al. (8) reported a unique, and genetically confirmed, pair of monozygotic twins in which one twin is affected with bilateral CLP and lip pits and the other is unaffected. With the use of genetic mapping and DNA sequencing, an *IRF6* mutation was identified in the affected twin and was absent in the unaffected co-twin, which confirmed *IRF6* as the first causal gene for VWS.

Later in 2011, another pair of monozygotic twins with VWS (25) was reported. In this case, both twins are affected with VWS, but despite carrying the same genetic mutation in *IRF6*, their phenotypes are markedly different, with one twin presenting bilateral cleft lip, cleft palate, and lower lip pits, while her twin sister has only lower lip pits. The variable expressivity of VWS phenotype and the phenotypic discordance even among monozygotic twins who carry the same mutation suggests the role of modifier factors, which can be genetic or epigenetic.

While MZ twins share identical DNA sequence, the fact that they can be phenotypically distinct offers important insights into the role of environmental factors. With the increased number of epigenetic studies, evidence suggests that epigenetic mechanisms may be potential mediators between environment and phenotypic expression.

DNA methylation of cytosines at CpG dinucleotides was first proposed as a mechanism of mammalian gene regulation in 1975 (3, 4), and has been since then the most broadly studied epigenetic mark. It occurs predominantly at the carbon-5 position of symmetrical CpG (cytosine and guanine separated by a phosphate) dinucleotides (5 mC). The state of DNA methylation is mitotically heritable through the activity of DNA methyltransferases (DNMTs), and it is essential for control of gene expression. Epigenetic marks like DNA methylation are essential for cell differentiation and preservation of tissue homogeneity. During development and throughout life, parent cells use epigenetic marks to ensure that their daughter cells will differentiate properly and function appropriately, and this message may persist through thousands of cell divisions for the lifetime of the organism, unless they are actively erased (by demethylase enzymes) or lost through epimutations. Typically, methylation of CpG sites in promoter regions of genes inhibits gene expression either due to the inability of specific transcription factors to bind methylated CpGs or the recruitment of methyl-CpG-binding proteins with transcription repression activity (26–28).

Aberrant DNA methylation patterns are universally recognized as playing an important role in human diseases, including monogenic and complex disorders. Recent studies indicate that abnormal methylation levels of key genes and/or regulatory elements are involved in heart disorders (29–34), depression and anxiety (35–42), several craniofacial syndromes (43–47), as well as nonsyndromic cleft lip and palate (48–53). In addition, several recent twin studies have shown additional evidence that DNA methylation may play an important role in phenotypic discordance (35, 48, 54–58).

In this article, we report the first DNA methylation profiles of monozygotic twins who present with discordant affection status and phenotypic expression for VWS. Twin pair 1 was previously reported in the study that discovered *IRF6* as the first causal gene for VWS (8). Even though they have been confirmed as monozygotic twins, one of them has VWS while the other is unaffected. This discordance in affection status is most likely a result of an early post-twinning mutation in the affected twin. Twin pair 2 was also previously reported (25), but both females are affected by VWS and carry the same mutation in *IRF6*. Despite being monozygotic and having the same mutation, their phenotype is markedly different.

Our goal is to explore the epigenetic contributions to VWS etiology by comparing the DNA methylation profiles of these two pairs of monozygotic discordant twins. While the mutation that causes VWS in these two twin pairs is known, additional mechanisms behind phenotypic risk and expression variability, especially in the second twin pair, remain underexplored.

## Material and methods

### Participants

**Figure 1:**
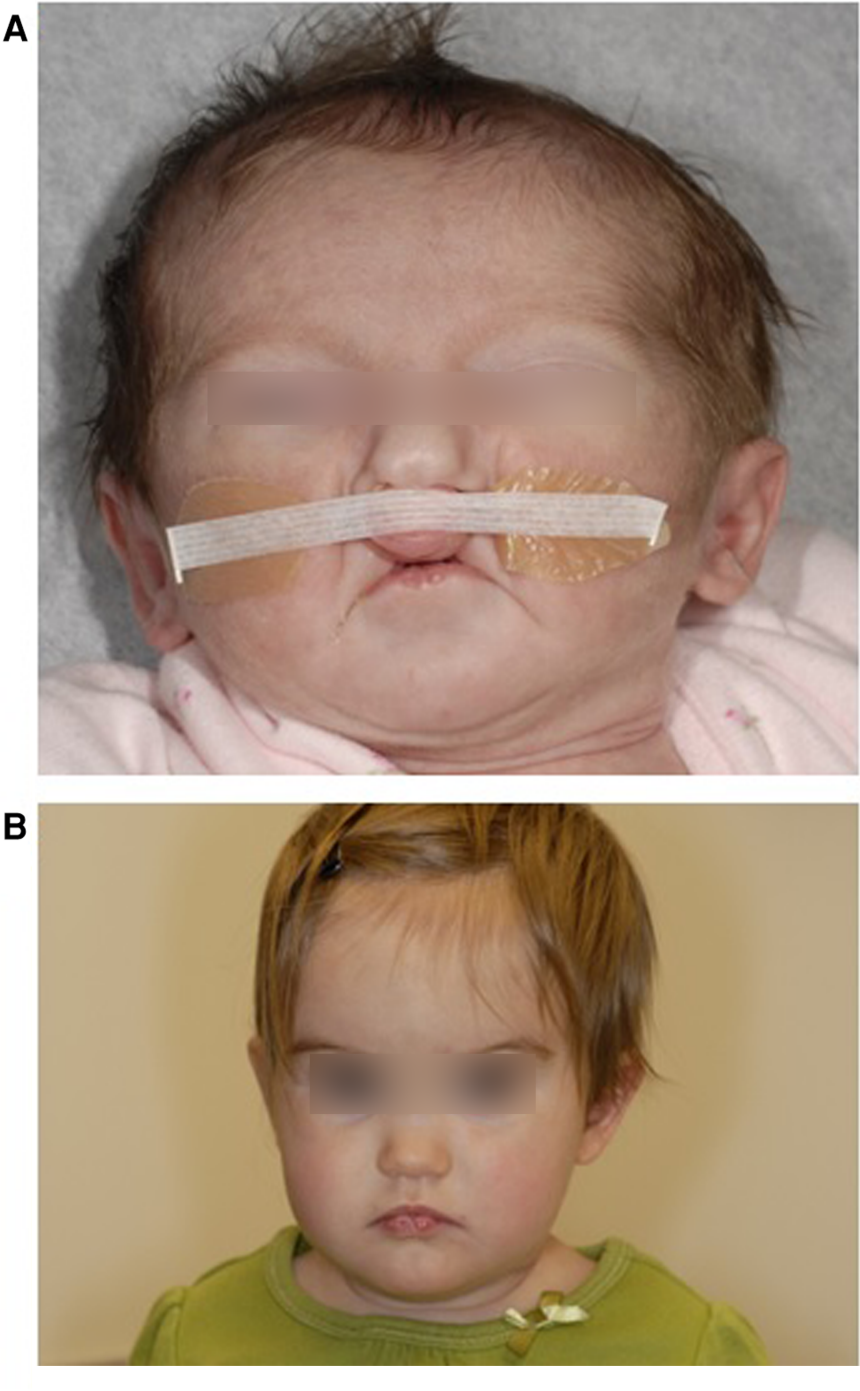
Twin 2A at 14 days showing bilateral paramedian lower lip pits and bilateral cleft lip. b: Twin 2B at 11 months showing bilateral paramedian lower lip pits. Reproduced with permission from Jobling et al. American Journal of Medical Genetics Part A 155:2008–2010.

We utilized DNA samples extracted from the whole blood of two pairs of monozygotic twins with VWS. For the purposes of this study, we will refer to these subjects as “twin pair 1” (8) (no photo available) and “twin pair 2” (25) (Figure 1).

In addition to the twins with VWS, we utilized existing epigenetic data from unaffected individuals as controls (*n* = 13 male controls for twin pair 1, and *n* = 12 female controls for twin pair 2), also obtained from whole blood samples. All samples have been collected as part of previous studies after approval by their respective IRBs and the signing of informed consent from parents or guardians.

### DNA sequencing

**Figure 2:**
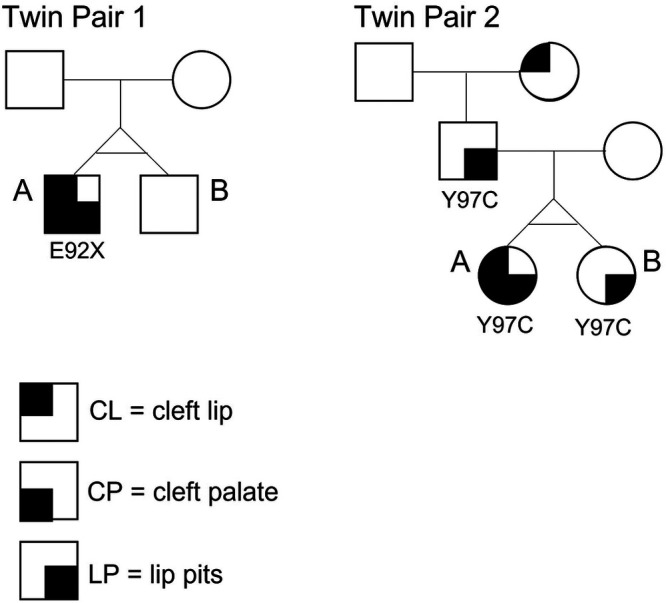
Pedigrees of the two monozygotic twin pairs in this study. Twin 1A is affected with CLP + LP and carries a nonsense mutation E92X in exon 4 of *IRF6*. The mutation is absent in Twin 1B. Twin 2A is affected with CLP + LP and carries a missense mutation Y97C, also located in exon 4 of *IRF6*. Twin 2B carries the same mutation but shows LP only (same as father).

DNA sequencing data were obtained from the respective previous studies (8, 25). For twin pair 1. Sanger sequencing of *IRF6* coding regions revealed a heterozygous *de novo* E92X nonsense mutation in exon 4 present in the affected twin. The mutation was absent in the unaffected twin and parents (Figure 2). For twin pair 2, Sanger sequencing of *IRF6* coding regions revealed that both twins and their affected father (lower lip pits) shared the same *IRF6* mutation, Y97C, also located in exon 4 (Figure 2).

### Sample quality and bisulfite conversion

DNA quality was assessed and quantified with DropSense96™ and Qubit™ dsDNA High Sensitivity Range Assay Kit (Thermo Fisher Scientific). After quantification of each specimen, 500 ng of genomic DNA was submitted to bisulfite conversion using the EZ DNA Methylation™ Kit (Zymo Research) according to the manufacturer's protocol.

### DNA methylation data

Genome-wide DNAm profiles were generated using Illumina's Infinium Methylation EPIC BeadChip assay (EPIC array) (Illumina, San Diego, CA, United States). The assay determines DNAm levels in more than 850,000 CpG sites and provides coverage of CpG islands, RefSeq genes, ENCODE open chromatin, ENCODE transcription factor-binding sites, and FANTOM5 enhancers. The assay was performed according to the manufacturer's instructions and scanned with the Illumina iScan System. To avoid batch effects, both members of each twin pair were assayed on the same array, and inter-batch duplicate samples were used as internal controls. As expected, duplicate samples showed high degrees of correlation (*r*^2 ^> 0.99).

### Data cleaning and preprocessing

Raw EPIC array data were preprocessed using ChAMP/RnBeads R/Bioconductor packages (59–61). Samples and probes that contained measurements with low technical quality (e.g., bad detection *P*-value) were excluded in the first step. Then, samples and probes with too many missing values or zero variability of their methylation values were eliminated next. Low-quality probes were removed using the Greedycut algorithm, based on a detection *P*-value threshold of 0.05, as implemented in the RnBeads package. Probes with less than three beads and probes with a missing value in at least 5% of the samples were also removed. Finally, probes that overlapped with known single nucleotide polymorphisms (SNPs) as assigned by the ChAMP per the version of dbSNP derived from Genome Reference Consortium Human Build 37 patch release 10 (GRCh37.p10) or that are located on sex chromosomes were also removed (59). The methylation level for each probe was measured as a beta value, calculated from the ratio of the methylated signals vs. the total sum of unmethylated and methylated signals, ranging between 0 (no methylation) and 1 (full methylation). This value was used for biological interpretation, visualization, and calculation of the absolute methylation difference (Δ*β* = |Twin1 *β*–Twin2 *β*|) of each pair separately. For twin pair A, which is a male pair, we used beta values of 13 male unaffected individuals to calculate the coefficient of variation in methylation levels of each CpG site after QC; as for twin pair B, we used beta values of 12 female unaffected individuals.

### Cell-type heterogeneity correction

We used the reference-based algorithm, EpiDISH (62), to perform an *in silico* deconvolution of the DNA methylation data. The package allows for dissection of intra-sample heterogeneity in EWAS and to infer the proportion of *a priori* known cell subtypes present in a mixture of cell types, such as in blood. The estimated cell-type composition was used in a logistic regression to correct for cell-type heterogeneity.

### Identification of differentially methylated positions (DMPs)

We selected differentially methylated positions (DMPs) based on the following criteria: (1) a coefficient of variation in methylation levels of unaffected individuals of less than 20%, and (2) intra-twin pair difference in methylation levels of at least 5% (Δ*β* > |0.05|). This strategy assumes that sites that exhibit larger inter-individual variation in methylation levels among the unaffected individuals are less likely to contribute to clefting. We used the DMP coordinates to annotate them to nearby genes and potential regulatory elements, such as known craniofacial enhancers that are active during early embryonic development (63). We then divided the DMPs into two subgroups for each pair for further analysis: (1) DMPs with higher methylation levels in twin A of each pair (Twin A > Twin B); and (2) DMPs with higher methylation levels in twin B of each pair (Twin B > Twin A). After annotating the DMPs to nearby genes based on genomic coordinates, we compared the list of genes to which the DMPs were annotated to a list of genes that contain transcription factor binding sites for *IRF6* (obtained from GeneCards, https://www.genecards.org/). In addition, we compared them to a list of genes previously associated with any type of OFCs (Supplementary Table S1).

### Gene ontology and pathways

We performed GO and enrichment analysis for the subgroups Twin A > Twin B and Twin B > Twin A separately using GREAT (http://great.stanford.edu) (64). We used a.bed file containing the genomic coordinates of all CpG sites interrogated that passed the initial QC (*n* = 735,653) as background.

## Results

Since the two twin pairs were of opposite gender and distinct ages, we analyzed both pairs separately with the same analytical pipeline and compared their results. Twin pair 1 is a male monozygotic twin pair; the affected twin (twin 1A) was shown to carry a nonsense mutation (E92X) in exon 4 of the *IRF6* gene, which was absent in the unaffected co-twin (twin 1B) and parents. The affected twin in this pair presented with cleft lip and cleft palate and bilateral lower lip pits. Twin Pair 2 is a female monozygotic twin pair; both girls and their affected father carry the same missense mutation (Y97C) in exon 4 of the *IRF6* gene. Although all three individuals share the same mutation, twin 2A presents with cleft lip and cleft palate and bilateral lower lip pits, while twin 2B and their father present only bilateral lower lip pits (no epigenetic data were generated for the father).

## Results for twin pair 1 (Twin 1a = CLP + lip pits; Twin 1b = unaffected)

**Figure 3:**
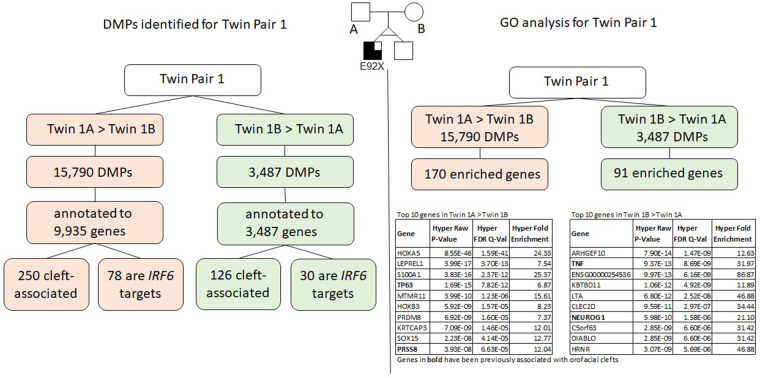
Summary of results for twin pair 1. Left side: DMPs identified for each group. Right side: results from the gene ontology and enrichment analysis for each group.

The results for twin pair 1 are shown below and summarized in Figure 3.

### Differentially methylated positions (DMPs)

We identified a total of 19,196 DMPs according to the inclusion criteria listed above; the DMPs were annotated to a total of 10,811 genes. Of all DMPs, 15,709 showed higher methylation levels (>5% difference) in twin 1A (Twin 1A > Twin 1B) and 3,487 showed higher methylation levels (>5% difference) in twin 1B (Twin 1B > Twin 1A); these were annotated to 9,935 and 3,702 genes, respectively. We found that 250 of the genes that contained DMPs in the Twin 1A > Twin 1B group and 126 genes in the Twin 1B > Twin 1A group have been associated with some type of OFC. Moreover, 78 out of the 9,935 genes that contained DMPs in the Twin 1A > Twin 1B group also contained a TFBS for *IRF6,* while, for the Twin 1B > Twin 1A group, 30 out of the 3,702 annotated genes were *IRF6* targets.

### Gene ontology analysis using GREAT

Gene ontology (GO) analysis for twin pair 1 returned a set of 170 genes enriched for the DMPs with >5% difference in methylation for Twin 1A > Twin 1B (Table 1). From these, 22 genes have been previously associated with some type of orofacial cleft (genes in bold in Table 1), with the *TP63* gene being the top cleft-associated hit (*P* = 7.82E^−12^). We repeated the same process with the DMPs from the Twin 1B > Twin 1A group and observed 91 genes enriched (Table 2), with 11 of them previously associated with orofacial clefts (genes in bold in Table 2). For this group, *TNF* (*P* = 8.69E-09) *and PAX7* (2.82E-03) were among the top hits.

**Table 1:** Gene ontology/enrichment results obtained from GREAT for Twin1A > Twin 1B.

Gene	Hyper raw *P*-value	Hyper FDR *Q*-value	Hyper fold enrichment
HOXA5	8.55E-46	1.59E-41	24.33
LEPREL1	3.99E-17	3.70E-13	7.54
S100A1	3.83E-16	2.37E-12	25.37
**TP63**	1.69E-15	7.82E-12	6.87
ZFP57	1.51E-14	5.61E-11	11.55
MTMR11	3.99E-10	1.23E-06	15.61
HOXB3	5.92E-09	1.57E-05	8.23
PRDM8	6.92E-09	1.60E-05	7.37
KRTCAP3	7.09E-09	1.46E-05	12.01
SOX15	2.23E-08	4.14E-05	12.77
**PRSS8**	3.93E-08	6.63E-05	12.04
WNT7B	4.24E-08	6.55E-05	3.77
ACTL7B	4.98E-08	7.10E-05	5.85
DSG1	6.67E-08	8.83E-05	11.39
LAMB3	9.25E-08	1.14E-04	9.37
LGALS8	1.30E-07	1.51E-04	6.41
DSC1	2.60E-07	2.84E-04	14.90
ST5	2.76E-07	2.84E-04	4.55
HOXA3	3.82E-07	3.73E-04	7.15
**KLF4**	4.13E-07	3.83E-04	4.68
PKP3	6.69E-07	5.91E-04	6.78
PTPN6	7.27E-07	6.13E-04	8.78
KLF10	1.02E-06	8.24E-04	4.65
RREB1	1.16E-06	8.93E-04	3.35
RPL27A	1.72E-06	1.27E-03	4.21
P4HB	1.85E-06	1.32E-03	6.89
FLRT3	2.04E-06	1.40E-03	5.51
EXPH5	2.05E-06	1.36E-03	7.81
PHB2	2.05E-06	1.36E-03	7.81
GSPT1	2.09E-06	1.29E-03	11.30
XRCC6BP1	2.12E-06	1.27E-03	4.39
CSF3	2.20E-06	1.27E-03	9.14
**GNE**	2.41E-06	1.35E-03	7.66
LRTOMT	2.41E-06	1.35E-03	7.66
**FGFR2**	2.44E-06	1.29E-03	3.56
**TGM1**	2.67E-06	1.38E-03	10.93
FXYD3	3.15E-06	1.58E-03	6.50
ADAMTS4	3.85E-06	1.88E-03	8.51
ANXA11	4.06E-06	1.93E-03	6.33
PPP1R27	4.06E-06	1.93E-03	6.33
HEATR1	4.59E-06	2.08E-03	6.24
ACTN4	4.59E-06	2.08E-03	6.24
**PPARA**	5.39E-06	2.33E-03	3.67
CTF1	6.55E-06	2.76E-03	9.64
NDUFS2	7.60E-06	3.13E-03	7.81
FGF5	8.69E-06	3.50E-03	4.44
TNKS1BP1	8.91E-06	3.52E-03	7.65
CLDN1	1.00E-05	3.86E-03	6.48
IL18BP	1.00E-05	3.86E-03	6.48
SEPT9	1.08E-05	4.00E-03	2.40
IPPK	1.15E-05	4.20E-03	5.64
WDR11	1.16E-05	4.14E-03	3.46
**GSTP1**	1.19E-05	4.15E-03	8.86
TACC2	1.19E-05	4.08E-03	4.32
TAF1B	1.22E-05	4.10E-03	4.64
KIFC3	1.26E-05	4.16E-03	4.02
**HOXB4**	1.51E-05	4.91E-03	4.95
FHL2	1.56E-05	4.99E-03	4.53
IDH2	1.56E-05	4.99E-03	4.53
ST3GAL4	1.62E-05	5.01E-03	3.37
SMG9	1.62E-05	4.94E-03	7.07
**GADD45A**	1.81E-05	5.41E-03	4.86
MCU	1.85E-05	5.44E-03	6.02
SLC47A2	1.85E-05	5.44E-03	6.02
LCA5	1.87E-05	5.34E-03	6.94
BCAS1	1.90E-05	5.35E-03	3.49
S100A2	1.91E-05	5.28E-03	10.41
LAPTM4A	1.98E-05	5.40E-03	4.81
**AZIN1**	2.30E-05	6.19E-03	4.06
FAM111B	2.38E-05	6.31E-03	10.04
DSG3	2.38E-05	6.31E-03	10.04
HOXB6	2.40E-05	6.19E-03	8.00
WBSCR27	2.83E-05	7.19E-03	7.81
**SCARB1**	3.17E-05	7.94E-03	2.87
IMP3	3.32E-05	8.20E-03	7.62
PPARGC1B	3.58E-05	8.75E-03	4.52
**ADIRF**	3.87E-05	9.33E-03	7.45
TAPBP	3.93E-05	9.35E-03	4.13
TPM4	4.11E-05	9.64E-03	6.24
ENC1	4.39E-05	1.02E-02	3.41
CLDN4	4.41E-05	1.01E-02	9.06
**KRT5**	5.32E-05	1.20E-02	8.78
**S100A10**	6.01E-05	1.34E-02	6.97
C6orf15	6.69E-05	1.48E-02	4.22
ECM2	6.91E-05	1.51E-02	6.83
POLR2L	6.91E-05	1.51E-02	6.83
NINJ2	7.95E-05	1.69E-02	3.60
CDCP2	8.23E-05	1.73E-02	5.68
ALDH3A1	8.23E-05	1.73E-02	5.68
CAPN12	8.23E-05	1.73E-02	5.68
CLSTN1	8.56E-05	1.75E-02	3.38
NBPF12	8.62E-05	1.74E-02	10.64
KRR1	8.96E-05	1.79E-02	3.56
KCNE4	8.96E-05	1.77E-02	4.46
NLRX1	9.03E-05	1.76E-02	6.56
**AQP3**	9.03E-05	1.74E-02	8.03
SREBF1	1.05E-04	2.00E-02	3.02
INPP5J	1.08E-04	2.05E-02	10.18
PHKG1	1.08E-04	2.05E-02	10.18
ABLIM1	1.13E-04	2.10E-02	3.48
ASB6	1.16E-04	2.14E-02	6.30
ALDH7A1	1.26E-04	2.29E-02	4.74
KAZN	1.26E-04	2.27E-02	2.53
YWHAQ	1.27E-04	2.27E-02	3.93
NCOR2	1.35E-04	2.38E-02	2.26
ARHGEF28	1.38E-04	2.42E-02	3.23
SMIM15	1.39E-04	2.40E-02	5.28
SLC39A2	1.45E-04	2.50E-02	7.39
OIT3	1.49E-04	2.53E-02	6.07
SFN	1.49E-04	2.53E-02	6.07
GRAMD4	1.55E-04	2.60E-02	3.84
PGRMC2	1.55E-04	2.60E-02	3.84
BTBD16	1.58E-04	2.60E-02	3.36
LY86	1.66E-04	2.70E-02	3.03
PHLDA1	1.66E-04	2.68E-02	3.56
**SLK**	1.67E-04	2.67E-02	5.96
KDELC2	1.67E-04	2.67E-02	5.96
NPTN	1.67E-04	2.67E-02	5.96
ZNF683	1.69E-04	2.63E-02	7.20
B4GALNT3	1.76E-04	2.72E-02	3.53
ERGIC1	1.78E-04	2.73E-02	4.11
OR5K1	1.86E-04	2.82E-02	23.42
**ARNTL**	1.91E-04	2.88E-02	2.99
NENF	1.91E-04	2.86E-02	4.48
VWF	1.91E-04	2.86E-02	4.48
TCAP	2.01E-04	2.95E-02	9.01
RERE	2.22E-04	3.24E-02	2.73
PINLYP	2.35E-04	3.40E-02	12.49
**JADE1**	2.35E-04	3.38E-02	3.43
SSPN	2.35E-04	3.38E-02	3.43
FAM50B	2.36E-04	3.34E-02	3.97
GSDMC	2.39E-04	3.36E-02	3.06
C1orf116	2.42E-04	3.37E-02	8.67
BAIAP2	2.42E-04	3.36E-02	2.62
MATN4	2.57E-04	3.53E-02	6.69
GLYATL1	2.57E-04	3.53E-02	6.69
MMP14	2.57E-04	3.53E-02	6.69
EIF4G1	2.61E-04	3.51E-02	5.56
GCOM1	2.68E-04	3.58E-02	4.80
B3GNT5	2.83E-04	3.75E-02	4.26
ENO1	2.90E-04	3.81E-02	5.46
TDRD10	2.93E-04	3.83E-02	6.53
**CD44**	2.97E-04	3.85E-02	3.15
C10orf95	3.08E-04	3.97E-02	11.71
SCGB1A1	3.09E-04	3.95E-02	3.84
AIFM2	3.21E-04	4.08E-02	5.37
AHNAK	3.29E-04	4.15E-02	4.17
TINAGL1	3.29E-04	4.15E-02	4.17
MTMR3	3.33E-04	4.15E-02	6.39
MFSD6L	3.33E-04	4.15E-02	6.39
S100A16	3.43E-04	4.22E-02	8.07
CERK	3.54E-04	4.32E-02	4.13
RAB27B	3.56E-04	4.31E-02	5.29
**SPRY1**	3.61E-04	4.35E-02	3.09
RLTPR	3.78E-04	4.52E-02	6.24
VARS2	3.80E-04	4.52E-02	4.09
PITPNA	3.80E-04	4.52E-02	4.09
IL1R2	3.83E-04	4.49E-02	3.25
S100A3	3.96E-04	4.62E-02	11.02
CALML3	4.00E-04	4.63E-02	3.72
PROSER2	4.09E-04	4.71E-02	4.05
ZFYVE21	4.09E-04	4.71E-02	4.05
TMPRSS2	4.17E-04	4.75E-02	3.04
RIN2	4.25E-04	4.80E-02	3.21
BOC	4.26E-04	4.79E-02	3.69
KLRG1	4.26E-04	4.76E-02	6.11
TMEM253	4.26E-04	4.76E-02	6.11
RBM28	4.33E-04	4.78E-02	5.12
PIK3C2G	4.33E-04	4.78E-02	5.12
**PVRL1**	4.42E-04	4.82E-02	2.57

Genes in bold have been previously associated with orofacial clefts.

**Table 2:** Gene ontology/enrichment results obtained from GREAT for Twin1B > twin 1A.

Gene	Hyper raw *P*-value	Hyper FDR *Q*-value	Hyper fold enrichment
ARHGEF10	7.90E-14	1.47E-09	12.63
**TNF**	9.37E-13	8.69E-09	31.97
ENSG00000254536	9.97E-13	6.16E-09	86.87
KBTBD11	1.06E-12	4.92E-09	11.89
LTA	6.80E-12	2.52E-08	46.88
CLEC2D	9.59E-11	2.97E-07	34.44
**NEUROG1**	5.98E-10	1.58E-06	21.10
C5orf63	2.85E-09	6.60E-06	31.42
DIABLO	2.85E-09	6.60E-06	31.42
HRNR	3.07E-09	5.69E-06	46.88
OR4E2	3.14E-09	5.29E-06	7.50
DAD1	3.56E-09	5.50E-06	7.43
RPTN	1.12E-08	1.60E-05	38.36
AURKC	1.94E-08	2.57E-05	58.60
CCR6	2.07E-07	2.56E-04	17.17
MTG1	2.62E-07	3.04E-04	16.59
C10orf76	2.68E-07	2.93E-04	23.01
RHOH	5.07E-07	5.23E-04	15.07
SOX2	7.51E-07	7.33E-04	7.96
CLEC2A	8.76E-07	8.13E-04	52.74
APOA1BP	9.17E-07	8.10E-04	28.51
ZC3H12D	1.86E-06	1.57E-03	16.66
TAS1R2	1.88E-06	1.51E-03	9.93
KCNIP2	2.70E-06	2.09E-03	15.63
**PAX7**	3.80E-06	2.82E-03	9.03
KCNG1	5.18E-06	3.69E-03	8.66
EPS15L1	5.34E-06	3.67E-03	13.91
RTP3	5.67E-06	3.75E-03	19.90
IQGAP3	6.82E-06	4.36E-03	19.18
APOBEC1	6.93E-06	4.29E-03	32.46
CLEC12A	6.93E-06	4.29E-03	32.46
LY9	8.87E-06	5.14E-03	18.19
PTGER4	9.10E-06	5.11E-03	9.78
RASIP1	9.42E-06	5.14E-03	30.14
UBXN11	1.09E-05	5.77E-03	29.10
NCMAP	1.23E-05	6.36E-03	17.01
ZNF365	1.43E-05	7.17E-03	11.72
RCAN3	1.44E-05	7.04E-03	16.48
PITPNC1	1.59E-05	7.55E-03	11.51
CD81	1.67E-05	7.75E-03	11.40
**ETS1**	1.71E-05	7.75E-03	7.34
ICOSLG	1.85E-05	8.17E-03	11.20
LAPTM5	2.25E-05	9.72E-03	10.82
CD163	2.35E-05	9.89E-03	24.11
ADO	2.37E-05	9.75E-03	10.73
NOL11	2.37E-05	9.75E-03	10.73
IKZF3	2.40E-05	9.45E-03	14.86
TSSC4	2.73E-05	1.05E-02	10.46
KLF2	2.99E-05	1.13E-02	10.29
SLC9A9	3.27E-05	1.21E-02	10.13
CCR7	3.55E-05	1.29E-02	13.70
C21orf33	3.58E-05	1.28E-02	9.97
B4GALNT4	4.02E-05	1.41E-02	13.35
BCL7A	4.81E-05	1.65E-02	12.86
LYNX1	4.89E-05	1.65E-02	20.09
PET112	5.33E-05	1.77E-02	6.25
SIT1	5.69E-05	1.85E-02	39.56
CLLU1	5.92E-05	1.89E-02	9.11
VEZT	6.74E-05	2.12E-02	11.99
RPS7	7.64E-05	2.36E-02	17.95
LGMN	7.78E-05	2.36E-02	8.67
CHST2	8.08E-05	2.42E-02	8.61
**IFITM3**	8.30E-05	2.44E-02	17.58
CMTM7	8.77E-05	2.54E-02	11.34
LRRC16A	8.92E-05	2.54E-02	6.84
**MYCN**	9.40E-05	2.64E-02	5.76
FAM26F	9.75E-05	2.70E-02	16.88
CCKBR	1.05E-04	2.88E-02	16.55
TDGF1	1.12E-04	3.02E-02	10.76
SLC43A2	1.12E-04	3.02E-02	10.76
**LRAT**	1.14E-04	2.97E-02	16.23
CDYL	1.15E-04	2.96E-02	6.56
DGKD	1.20E-04	3.05E-02	8.01
**NR2F2**	1.20E-04	3.02E-02	4.89
TH	1.42E-04	3.51E-02	10.24
PITPNA	1.42E-04	3.51E-02	10.24
SLMO2	1.52E-04	3.66E-02	15.07
PRKCDBP	1.52E-04	3.66E-02	15.07
SPIN1	1.74E-04	4.09E-02	14.55
ZNF80	1.76E-04	4.07E-02	27.52
DMRT2	1.77E-04	4.06E-02	9.77
MMP7	1.86E-04	4.21E-02	14.30
**RRAGC**	1.89E-04	4.22E-02	6.05
ENSG00000250264	2.12E-04	4.68E-02	13.83
SERPINB9	2.26E-04	4.93E-02	13.61
FSTL3	2.26E-04	4.88E-02	25.32
CHRNA9	2.28E-04	4.87E-02	9.25
**GNG4**	2.38E-04	5.01E-02	9.17
SERPINB1	2.40E-04	5.01E-02	13.39
**SNX9**	2.41E-04	4.97E-02	5.81

Genes in bold have been previously associated with orofacial clefts.

## Results for twin pair 2 (Twin 2a = CLP + lip pits; Twin 2b = lip pits)

**Figure 4:**
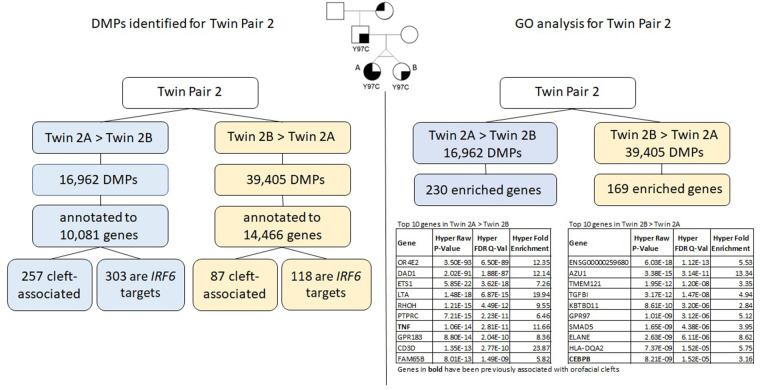
Summary of results for twin pair 2. Left side: DMPs identified for each group. Right side: results from the gene ontology and enrichment analysis for each group.

The results for twin pair 2 are explained below and summarized in Figure 4.

### Differentially methylated positions (DMPs)

We identified a total of 56,367 DMPs according to the inclusion criteria listed above (Δ*β* > |0.05|), and the DMPs were annotated to a total of 15,424 genes. Of all DMPs, 16,962 showed higher methylation levels in twin 2A (Twin 2A > Twin 2B) and 39,405 showed higher methylation levels in twin 2B (Twin 2B > Twin 2A); these were annotated to 10,081 and 14.466 genes, respectively. We found that 257 of the genes that contained DMPs in the Twin 2A > Twin 2B group and 87 genes in the Twin 2B > Twin 2A group have been associated with some type of OFC. Moreover, 303 out of the 10,081 genes that contained DMPs in the Twin 2A > Twin 2B group also contained a TFBS for *IRF6,* while, for the Twin 2B > Twin 2A group, 118 out of the 14,466 annotated genes that contained DMPs were *IRF6* targets.

### Gene ontology analysis using GREAT

Gene ontology (GO) analysis for twin pair 2 returned a set of 230 genes enriched for the DMPs with >5% difference in methylation for Twin 2A > Twin 2B (Table 3). From these, six genes have been previously associated with some type of orofacial cleft (genes in bold in Table 3). We repeated the same process with the DMPs from the Twin 2B > Twin 2A group and observed 169 genes enriched (Table 4), with 4 of them previously associated with orofacial clefts (genes in bold in Table 4). For twin pair 2, cleft-associated genes like *TNF* (*P* = 2.81E-11) *and ARID5B* (1.73E-07) were among the top hits.

**Table 3:** Gene ontology/enrichment results obtained from GREAT for Twin2A > Twin 2B.

Gene	Hyper raw *P*-value	Hyper FDR *Q*-value	Hyper fold enrichment
OR4E2	3.50E-93	6.50E-89	12.35
DAD1	2.02E-91	1.88E-87	12.14
ETS1	5.85E-22	3.62E-18	7.26
LTA	1.48E-18	6.87E-15	19.94
RHOH	1.21E-15	4.49E-12	9.55
PTPRC	7.21E-15	2.23E-11	6.46
**TNF**	1.06E-14	2.81E-11	11.66
GPR183	8.80E-14	2.04E-10	8.36
CD3D	1.35E-13	2.77E-10	23.87
FAM65B	8.01E-13	1.49E-09	5.82
RTKN2	2.73E-12	4.60E-09	7.06
TM9SF2	1.38E-11	2.13E-08	6.51
SATB1	2.34E-11	3.34E-08	6.00
LRRC16A	4.15E-11	5.50E-08	5.09
CCR9	9.48E-11	1.17E-07	12.70
COA1	1.41E-10	1.63E-07	10.85
**ARID5B**	1.59E-10	1.73E-07	4.98
CD3G	2.11E-10	2.17E-07	24.80
IKZF1	2.99E-10	2.92E-07	6.20
FOXP1	4.87E-10	4.51E-07	3.67
RFTN1	5.43E-10	4.79E-07	6.83
ITPKB	1.15E-09	9.73E-07	5.42
CD28	3.61E-09	2.91E-06	7.69
OXNAD1	5.29E-09	4.09E-06	6.85
CXCR6	7.44E-09	5.52E-06	8.98
CABYR	8.20E-09	5.85E-06	4.63
PHLPP1	1.28E-08	8.79E-06	4.01
UBASH3A	1.58E-08	1.05E-05	9.55
ITK	1.69E-08	1.08E-05	13.02
STK17B	2.45E-08	1.51E-05	6.14
LCLAT1	2.56E-08	1.53E-05	5.69
CTLA4	3.03E-08	1.75E-05	9.01
ARL4C	3.37E-08	1.89E-05	4.10
TMEM75	3.97E-08	2.17E-05	3.45
C1orf95	4.03E-08	2.14E-05	4.88
CHI3L2	4.13E-08	2.13E-05	7.77
FAM71B	6.26E-08	3.14E-05	23.67
TNFAIP8	7.45E-08	3.64E-05	5.66
BCL11B	8.13E-08	3.87E-05	3.52
TBC1D5	8.77E-08	4.07E-05	4.21
SLC30A5	9.67E-08	4.37E-05	4.39
TRIM69	9.80E-08	4.33E-05	10.85
GIMAP7	1.19E-07	5.14E-05	12.86
FYN	1.23E-07	5.17E-05	4.54
TCF7	1.23E-07	5.07E-05	6.41
CXCR5	1.40E-07	5.65E-05	7.83
SPP2	1.51E-07	5.97E-05	4.73
LAMA3	1.74E-07	6.71E-05	4.44
BCL2	1.74E-07	6.57E-05	3.88
LHX6	1.76E-07	6.54E-05	6.85
LBH	1.93E-07	7.02E-05	5.68
CLEC2D	2.27E-07	8.09E-05	8.51
CD247	3.80E-07	1.33E-04	5.01
KCNH8	3.82E-07	1.31E-04	5.38
ATP6V1G3	4.14E-07	1.40E-04	6.35
LCK	4.43E-07	1.47E-04	7.02
C2orf40	5.03E-07	1.64E-04	4.13
BTG1	5.64E-07	1.80E-04	4.31
PRKCH	6.34E-07	1.99E-04	4.07
**KLF12**	6.39E-07	1.97E-04	3.72
SLMO2	6.76E-07	2.06E-04	7.61
IKZF3	6.95E-07	2.08E-04	6.72
VCPKMT	8.89E-07	2.62E-04	5.02
GMPR	1.17E-06	3.40E-04	3.73
GPR126	1.30E-06	3.71E-04	4.55
HIF1A	1.44E-06	4.04E-04	3.68
**PIK3R1**	1.47E-06	4.07E-04	3.19
B2M	1.54E-06	4.21E-04	11.68
ATXN1	1.59E-06	4.27E-04	3.51
CD2	2.07E-06	5.49E-04	6.04
IL19	2.09E-06	5.47E-04	9.14
EIF4E3	2.13E-06	5.48E-04	3.58
IFNG	2.32E-06	5.90E-04	5.42
FAM69A	2.35E-06	5.89E-04	5.97
IL1R2	2.67E-06	6.60E-04	4.06
ASTL	2.67E-06	6.52E-04	19.72
ARF6	2.78E-06	6.69E-04	4.57
FAM208A	3.29E-06	7.83E-04	4.23
TRAF3IP2	3.35E-06	7.85E-04	3.99
FASLG	3.58E-06	8.31E-04	5.21
SP140	4.40E-06	1.01E-03	10.13
ZBED2	4.40E-06	1.01E-03	10.13
PATL2	4.49E-06	1.00E-03	18.08
BCL2L1	5.20E-06	1.15E-03	6.97
PDE7A	5.42E-06	1.18E-03	5.49
RNGTT	5.44E-06	1.17E-03	4.62
HECW2	6.07E-06	1.29E-03	5.42
DOCK10	7.12E-06	1.50E-03	4.51
RASGRP1	7.19E-06	1.50E-03	3.18
IL12RB2	8.90E-06	1.83E-03	5.79
COG6	9.83E-06	2.00E-03	3.35
KLF3	1.00E-05	2.02E-03	3.22
HIVEP2	1.05E-05	2.09E-03	4.08
ZNF80	1.08E-05	2.13E-03	11.32
FIGNL1	1.09E-05	2.13E-03	4.34
ZNF831	1.13E-05	2.18E-03	5.64
CCR7	1.13E-05	2.18E-03	5.64
IL7R	1.17E-05	2.21E-03	4.65
CUL3	1.31E-05	2.46E-03	4.00
GPR18	1.32E-05	2.44E-03	7.23
LIME1	1.41E-05	2.59E-03	10.85
CD3E	1.41E-05	2.59E-03	10.85
UPF3A	1.60E-05	2.88E-03	8.44
COX4I2	1.60E-05	2.86E-03	6.10
IL6ST	1.80E-05	3.19E-03	3.09
ARHGDIB	1.82E-05	3.18E-03	10.41
FOXO1	1.94E-05	3.36E-03	3.19
LPP	2.10E-05	3.61E-03	2.64
RAG1	2.29E-05	3.90E-03	13.56
RHBDD1	2.64E-05	4.45E-03	5.74
KLRD1	2.77E-05	4.63E-03	7.79
CHIA	2.79E-05	4.62E-03	6.55
HIVEP3	2.81E-05	4.62E-03	3.10
DCN	3.03E-05	4.94E-03	3.94
RNF19A	3.30E-05	5.33E-03	3.68
MTFR1	3.34E-05	5.35E-03	5.58
GIMAP5	3.89E-05	6.17E-03	7.41
TNRC6B	4.30E-05	6.76E-03	3.59
NR5A2	4.39E-05	6.85E-03	3.11
GIMAP4	4.50E-05	6.96E-03	8.98
GJA10	5.00E-05	7.67E-03	3.76
ID3	5.23E-05	7.96E-03	5.28
OR10T2	5.30E-05	7.99E-03	17.36
RORA	5.41E-05	8.10E-03	3.06
FCRL3	5.52E-05	8.19E-03	8.68
PCDHGA5	5.52E-05	8.19E-03	8.68
ICOS	5.96E-05	8.71E-03	4.67
SEMA4D	5.96E-05	8.71E-03	4.67
ANKRD44	6.05E-05	8.70E-03	3.94
TIGIT	6.18E-05	8.81E-03	5.88
RCAN2	6.18E-05	8.81E-03	5.88
MMP7	6.18E-05	8.81E-03	5.88
MMP20	6.25E-05	8.71E-03	6.90
CHRNA9	6.52E-05	9.02E-03	4.22
NCK2	6.53E-05	8.97E-03	3.29
**RPL5**	6.98E-05	9.52E-03	5.79
CNR1	7.06E-05	9.57E-03	4.19
MYL4	7.54E-05	1.01E-02	10.85
BACH2	7.55E-05	1.01E-02	3.41
LZTFL1	8.10E-05	1.07E-02	8.14
SPG11	8.38E-05	1.10E-02	6.60
STRBP	8.79E-05	1.15E-02	4.94
CD83	9.26E-05	1.20E-02	2.65
STARD4	9.69E-05	1.25E-02	3.53
STK17A	9.69E-05	1.25E-02	3.53
ZCCHC7	9.70E-05	1.23E-02	4.88
SOX4	1.01E-04	1.27E-02	2.64
CLLU1	1.07E-04	1.34E-02	3.72
COTL1	1.07E-04	1.33E-02	4.82
TLR2	1.10E-04	1.37E-02	4.34
WIPI2	1.21E-04	1.49E-02	3.94
TMEM161B	1.23E-04	1.50E-02	2.60
UBE2E3	1.24E-04	1.51E-02	3.44
AKAP13	1.25E-04	1.50E-02	2.76
MRPS10	1.30E-04	1.56E-02	4.25
TNFSF11	1.32E-04	1.57E-02	3.42
DNTT	1.37E-04	1.62E-02	7.44
MUC4	1.40E-04	1.65E-02	3.40
SPATA13	1.49E-04	1.74E-02	3.38
TAP1	1.55E-04	1.80E-02	4.59
OPRM1	1.59E-04	1.83E-02	3.57
NPIPB11	1.64E-04	1.88E-02	5.96
CCR6	1.70E-04	1.94E-02	4.54
COL4A4	1.71E-04	1.94E-02	5.11
SUSD1	1.79E-04	2.02E-02	4.09
PTTG2	1.81E-04	2.02E-02	3.00
ANKRD46	1.81E-04	2.01E-02	3.52
LTB	1.86E-04	2.05E-02	5.84
PM20D1	1.86E-04	2.05E-02	5.84
POU5F1	1.86E-04	2.03E-02	3.76
IFITM1	1.91E-04	2.08E-02	9.04
NDUFA8	2.03E-04	2.19E-02	4.44
ENSG00000243627	2.10E-04	2.25E-02	5.73
RUFY3	2.10E-04	2.25E-02	5.73
SKP1	2.10E-04	2.25E-02	5.73
PRKCQ	2.35E-04	2.48E-02	2.62
PGAP1	2.43E-04	2.55E-02	3.94
PDCD4	2.44E-04	2.54E-02	3.64
RGS13	2.54E-04	2.63E-02	6.68
CAMK4	2.56E-04	2.64E-02	4.82
**CHD7**	2.57E-04	2.63E-02	2.79
POU2F1	2.61E-04	2.66E-02	3.38
IL10	2.65E-04	2.69E-02	5.52
PTP4A2	2.82E-04	2.84E-02	4.76
DENND2D	2.93E-04	2.94E-02	6.51
TESPA1	2.93E-04	2.94E-02	6.51
EDN2	3.00E-04	2.97E-02	2.65
ITGB2	3.09E-04	3.05E-02	4.69
MUC20	3.09E-04	3.05E-02	4.69
HOXA5	3.09E-04	3.05E-02	4.69
TRAT1	3.14E-04	3.05E-02	11.57
C4orf26	3.14E-04	3.05E-02	11.57
EVL	3.28E-04	3.15E-02	3.12
TMPPE	3.32E-04	3.18E-02	5.33
IFNK	3.37E-04	3.20E-02	6.35
GRB7	3.39E-04	3.21E-02	4.63
TRERF1	3.48E-04	3.28E-02	3.77
BTLA	3.70E-04	3.47E-02	5.24
NFATC3	3.72E-04	3.46E-02	4.57
DRD3	3.85E-04	3.57E-02	6.20
SLFN5	3.85E-04	3.57E-02	6.20
CCR2	4.10E-04	3.76E-02	7.75
ATG3	4.10E-04	3.76E-02	7.75
KLF13	4.25E-04	3.87E-02	3.03
ZAP70	4.32E-04	3.91E-02	3.41
IPCEF1	4.32E-04	3.91E-02	3.41
PLCL2	4.38E-04	3.93E-02	3.19
UNC50	4.39E-04	3.91E-02	6.05
PLEKHG7	4.58E-04	4.06E-02	3.65
KDM8	4.63E-04	4.09E-02	3.18
SGIP1	4.71E-04	4.14E-02	3.00
SEPT9	4.78E-04	4.18E-02	2.01
MANBA	4.89E-04	4.26E-02	3.62
TAF8	4.90E-04	4.25E-02	3.94
ENPP5	5.06E-04	4.37E-02	4.98
HDAC1	5.06E-04	4.37E-02	4.98
HUS1B	5.17E-04	4.42E-02	3.34
SP100	5.27E-04	4.48E-02	4.34
CCNI	5.27E-04	4.48E-02	4.34
SIT1	5.27E-04	4.45E-02	10.21
THBS1	5.48E-04	4.60E-02	2.31
NCMAP	5.59E-04	4.67E-02	4.90
ZFP36L1	5.60E-04	4.66E-02	2.16
AKAP11	5.68E-04	4.70E-02	3.87
NINJ2	6.05E-04	4.99E-02	3.08
DYNLRB2	6.09E-04	5.00E-02	2.79
NEK7	6.10E-04	4.98E-02	3.83
**MAF**	6.10E-04	4.97E-02	2.42
MTA3	6.17E-04	4.99E-02	4.82
RCAN3	6.17E-04	4.99E-02	4.82

Genes in bold have been previously associated with orofacial clefts.

**Table 4:** Gene ontology/enrichment results obtained from GREAT for Twin2B > Twin 2A.

Gene	Hyper raw *P*-value	Hyper FDR *Q*-value	Hyper fold enrichment
ENSG00000259680	6.03E-18	1.12E-13	5.53
AZU1	3.38E-15	3.14E-11	13.34
TMEM121	1.95E-12	1.20E-08	3.35
TGFBI	3.17E-12	1.47E-08	4.94
KBTBD11	8.61E-10	3.20E-06	2.84
GPR97	1.01E-09	3.12E-06	5.12
SMAD5	1.65E-09	4.38E-06	3.95
ELANE	2.63E-09	6.11E-06	8.62
HLA-DQA2	7.37E-09	1.52E-05	5.75
**CEBPB**	8.21E-09	1.52E-05	3.16
PPP1R27	8.55E-09	1.44E-05	4.80
CFD	1.00E-08	1.55E-05	5.64
PRTN3	1.34E-08	1.92E-05	8.56
MPO	1.41E-08	1.86E-05	6.38
TRPM2	1.76E-08	2.17E-05	5.12
OMA1	1.79E-08	2.07E-05	3.54
MGRN1	2.94E-08	3.21E-05	3.21
ZFP57	5.20E-08	5.36E-05	4.54
JARID2	5.71E-08	5.57E-05	2.20
EMILIN1	6.80E-08	6.31E-05	11.49
PTPN1	7.01E-08	6.19E-05	2.62
SPI1	9.05E-08	7.63E-05	6.07
ANKRD13D	1.01E-07	8.11E-05	5.56
OSBPL5	2.02E-07	1.56E-04	3.24
CLDN15	2.90E-07	2.15E-04	6.06
NUDT16L1	3.09E-07	2.20E-04	3.60
PWWP2B	4.10E-07	2.82E-04	2.79
FAM92B	4.51E-07	2.99E-04	2.00
MKRN3	9.47E-07	6.06E-04	7.64
C2orf62	1.10E-06	6.81E-04	4.38
NINJ2	1.31E-06	7.82E-04	2.87
FXYD1	1.48E-06	8.59E-04	7.31
CPEB4	1.58E-06	8.88E-04	2.67
**ASXL1**	1.96E-06	1.04E-03	3.78
P4HB	3.04E-06	1.56E-03	4.06
CARS	3.88E-06	1.94E-03	2.92
AMZ1	3.89E-06	1.90E-03	2.46
**DAB1**	4.33E-06	2.06E-03	2.64
RUSC2	4.89E-06	2.27E-03	4.15
BOD1	7.55E-06	3.33E-03	2.43
PRKCD	7.58E-06	3.27E-03	3.79
NFE2	9.80E-06	4.13E-03	6.00
LTC4S	9.92E-06	4.09E-03	4.48
C20orf112	1.03E-05	4.15E-03	3.11
NDRG1	1.06E-05	4.18E-03	2.57
ENG	1.17E-05	4.51E-03	4.12
ALG10	1.18E-05	4.49E-03	2.74
ZNF385A	1.25E-05	4.64E-03	3.85
GPR84	1.42E-05	5.16E-03	4.05
SCARB1	1.44E-05	5.14E-03	2.13
TMEM105	1.49E-05	5.21E-03	2.16
RHOU	1.68E-05	5.76E-03	2.41
IFITM10	1.71E-05	5.78E-03	3.98
PRAM1	1.71E-05	5.78E-03	3.98
**DUSP22**	1.74E-05	5.66E-03	3.11
RNASEH2C	1.84E-05	5.89E-03	5.60
ZSCAN10	1.88E-05	5.90E-03	4.23
DTNBP1	1.94E-05	6.01E-03	2.18
TMEM78	1.99E-05	6.05E-03	2.65
METTL22	2.06E-05	6.17E-03	3.92
ABCC1	2.18E-05	6.42E-03	2.96
B4GALT5	2.19E-05	6.35E-03	3.32
CRIM1	2.20E-05	6.27E-03	2.64
S100A8	2.37E-05	6.66E-03	7.26
URAD	2.40E-05	6.64E-03	4.47
USP32	2.76E-05	7.53E-03	3.42
ATP11B	2.89E-05	7.76E-03	2.43
HLA-DQB2	2.92E-05	7.73E-03	3.58
ANPEP	2.95E-05	7.70E-03	3.79
RREB1	3.06E-05	7.87E-03	2.11
SF3A3	3.27E-05	8.32E-03	5.25
FXYD7	3.27E-05	8.32E-03	5.25
RPH3AL	3.50E-05	8.55E-03	2.36
RAB24	3.68E-05	8.87E-03	4.28
TOMM20	3.71E-05	8.82E-03	2.20
PAX8	3.83E-05	8.98E-03	3.18
B3GNT8	4.40E-05	1.02E-02	5.75
OR4C12	4.40E-05	1.02E-02	5.75
B4GALNT3	4.44E-05	1.00E-02	2.58
ROPN1L	4.80E-05	1.07E-02	2.36
SERTAD2	4.97E-05	1.10E-02	2.45
FEZ2	4.98E-05	1.09E-02	2.71
DOC2B	5.12E-05	1.11E-02	2.39
HAAO	5.25E-05	1.12E-02	2.30
ARHGEF10	5.90E-05	1.24E-02	2.12
GJD4	6.45E-05	1.33E-02	3.05
ABAT	6.45E-05	1.33E-02	3.05
SPIDR	6.46E-05	1.30E-02	2.41
LY86	6.57E-05	1.31E-02	2.24
STX2	7.20E-05	1.42E-02	2.30
HLA-DRB5	7.23E-05	1.41E-02	4.34
SRR	7.50E-05	1.45E-02	2.71
GPR21	7.89E-05	1.51E-02	3.47
P2RY2	8.37E-05	1.58E-02	3.27
VPREB1	8.84E-05	1.66E-02	2.86
DOK3	9.05E-05	1.68E-02	4.67
LPO	9.05E-05	1.68E-02	4.67
ABCC6	9.35E-05	1.70E-02	2.96
F2RL2	9.62E-05	1.73E-02	3.23
VILL	9.64E-05	1.72E-02	3.08
PSMA6	9.64E-05	1.72E-02	3.08
PDLIM7	9.70E-05	1.70E-02	3.88
CORO2A	9.70E-05	1.70E-02	3.88
AGAP2	9.70E-05	1.70E-02	3.88
FHL3	1.05E-04	1.79E-02	5.94
NNAT	1.11E-04	1.87E-02	2.56
LRG1	1.14E-04	1.90E-02	4.54
MYBPHL	1.14E-04	1.90E-02	4.54
AOAH	1.14E-04	1.87E-02	2.13
ZNF727	1.14E-04	1.86E-02	3.56
ASGR2	1.14E-04	1.86E-02	3.56
PHYKPL	1.21E-04	1.94E-02	2.19
LRRC3	1.24E-04	1.96E-02	2.79
MTRNR2L1	1.24E-04	1.96E-02	2.79
ELMO3	1.36E-04	2.12E-02	4.98
NCOA4	1.42E-04	2.20E-02	4.42
PCDHB4	1.44E-04	2.21E-02	5.68
SNCG	1.44E-04	2.21E-02	5.68
ZNRF2	1.54E-04	2.33E-02	2.65
PCDHA9	1.55E-04	2.31E-02	8.49
NRROS	1.56E-04	2.31E-02	3.45
SYT12	1.56E-04	2.31E-02	3.45
P2RY12	1.60E-04	2.34E-02	3.97
UACA	1.74E-04	2.52E-02	2.05
CTSG	1.74E-04	2.51E-02	6.59
TCHH	1.75E-04	2.49E-02	4.82
PCDHA10	1.75E-04	2.49E-02	4.82
TIMM23	1.75E-04	2.49E-02	4.82
TACSTD2	1.75E-04	2.49E-02	4.82
ZNF710	1.86E-04	2.56E-02	3.04
NLRP3	1.93E-04	2.63E-02	3.60
PILRA	1.93E-04	2.61E-02	3.89
TKT	2.11E-04	2.82E-02	3.01
MTMR7	2.15E-04	2.85E-02	3.15
TSPAN18	2.16E-04	2.84E-02	2.23
LGI4	2.16E-04	2.83E-02	4.20
PVRL4	2.22E-04	2.88E-02	4.67
PTPN12	2.26E-04	2.92E-02	3.54
ALAS1	2.31E-04	2.95E-02	3.81
CCDC155	2.49E-04	3.17E-02	6.23
ST18	2.52E-04	3.18E-02	2.55
CD164	2.53E-04	3.17E-02	2.83
CCDC146	2.53E-04	3.15E-02	7.78
TGFB1I1	2.64E-04	3.27E-02	4.10
HOXB8	2.64E-04	3.27E-02	4.10
HLA-DPA1	2.66E-04	3.24E-02	2.06
CEP57L1	2.70E-04	3.27E-02	2.94
WISP1	2.77E-04	3.34E-02	2.53
P2RX1	2.80E-04	3.35E-02	3.07
ELMO1	2.82E-04	3.35E-02	2.15
PSD4	2.88E-04	3.40E-02	2.69
LYZL2	2.90E-04	3.41E-02	2.45
NFIC	3.01E-04	3.51E-02	2.27
LYNX1	3.20E-04	3.71E-02	4.00
SLC23A2	3.21E-04	3.70E-02	3.20
P2RY13	3.48E-04	3.99E-02	5.90
ZNF19	3.48E-04	3.99E-02	5.90
ANTXRL	3.48E-04	3.94E-02	4.40
ERG	3.51E-04	3.95E-02	2.25
C19orf77	3.53E-04	3.95E-02	2.64
NUP210L	3.84E-04	4.24E-02	3.59
POMZP3	3.93E-04	4.32E-02	7.18
**SLC19A1**	4.16E-04	4.54E-02	2.32
ARL5C	4.31E-04	4.67E-02	4.27
PPARGC1B	4.32E-04	4.66E-02	2.60
ANGPT2	4.32E-04	4.66E-02	2.60
CEBPE	4.51E-04	4.80E-02	3.52
MAML1	4.62E-04	4.90E-02	3.82

Genes in bold have been previously associated with orofacial clefts.

## Discussion

Monogenic diseases, like VWS, often feature highly variable phenotypes, despite a usually well-defined genetic cause for the disease. Modifier genes and epigenetic mechanisms are believed to influence phenotypic risk and variable expressivity. In this article, we see the rare event of monozygotic twins carrying the same mutation (twin pair 2), who display extremely divergent phenotypes for VWS.

Monozygotic twins arise from the same fertilized oocyte and are, therefore, believed to have the same DNA sequence. Postzygotic mutations are responsible for a substantial proportion of *de novo* mutations in humans and have been shown to contribute to disease phenotypic variability, including among twins (65–71). In twins, the timing of postzygotic mutations determines whether they are present in both twins (pre-twinning mutation) or in only one twin (post-twinning mutation) (68). Our twin pair 1 is an example of post-twinning mutation, where only twin 1A became a carrier of the *IRF6* mutation, while twin 1B does not have the causal mutation and is, therefore, unaffected. In twin pair 2, both females inherited the *IRF6* mutation from their affected father (Figure 2).

In addition to playing an essential role in estimating phenotypic variability, twin studies offer an opportunity to study epigenetic variation as a quantitative trait. The monozygotic twin discordance rate observed in OFCs (≥50%) suggests that unexplained environmental and epigenetic factors play an etiological role. Epigenetic studies have shown that changes in DNA methylation play a role in nonsyndromic (48–51, 53, 72, 73) and syndromic forms of OFCs. Among the cleft syndromes most commonly associated with differences in DNA methylation are Kabuki and Charge syndromes (43, 44, 46, 47, 74, 75), both of which have specific DNA methylation signatures. We describe the first whole genome methylation profiling of MZ twins discordant for VWS and discuss the possible roles that differences in DNAm may have in phenotypic discordance.

Gene ontology and enrichment analysis of the thousands of CpG sites with differential methylation identified for each pair revealed a list of enriched genes that included, but were not limited to, known cleft-associated genes. The top genes that showed different levels of DNAm between each twin pair include genes like *HOXA5, LEPREL1, ARHGEF10, OR4E2, DAD1, AZU1,* and *TP63,* among others (please refer to Tables 1–4). From these, *TP63* is known to cause syndromic forms of OFCs and is associated with nonsyndromic clefts; in addition, *TP63* is directly involved in the activation of *IRF6*.

Mutations in the gene encoding the transcription factor interferon regulatory factor 6 (*IRF6)*, cause VWS and PPS, both characterized by ectodermal anomalies and CL/P or CP; mutations in the transcription factor p63 cause autosomal dominant ectodermal dysplasia syndromes such as ectrodactyly ectodermal dysplasia-clefting (EEC), which is also characterized by CL/P or CP and defects in ectoderm-derived tissues, such as the epidermis, hair, teeth, and glands (76). Moreover, mutations in both genes have been implicated in nonsyndromic OFCs (77).

Studies have shown that *IRF6* is transcriptionally activated by *TP63* and, in turn, induces the *TP63* proteasome-mediated downregulation, thereby limiting epithelial cell proliferative potential (78), including in the medial epithelial seam around the time of palate closure. Such process is critical for normal palatal fusion (79–82).

Among the cleft-associated genes in our dataset, *TP63* was the most significant hit (*P* = 7.82E-12). Both twin pairs presented differential DNAm levels in CpG sites in/near *TP63* (Twin 1A > Twin 1B and Twin 2A < Twin 2B). Interestingly, in twin pair 2, the twin with only lip pits (twin 2B) presents higher levels of methylation in the promoter region of the *TP63* gene. It has been shown that *TP63* downregulation necessitates normal IRF6 function, and that this task is absent in *irf6* mutant mice, who remain p63 positive in the palatal medial edge epithelial (83). Given that *TP63* and *IRF6* work together in a regulatory loop to coordinate epithelial proliferations, it is plausible that DNAm can modify the effects of the etiological variant and lead to markedly distinct phenotypes, even among individuals carrying the same mutation. Since methylation of promoters is often associated with gene silencing, it is possible that this epigenetic mechanism compensates for the inability of the mutated *IRF6* to downregulate *TP63*, therefore contributing to a less severe phenotype in twin 2B.

There are some limitations in our study. First, our study includes two pairs of MZ twins discordant for affection status and phenotype, but given the rarity of the condition, we consider the presented data of importance to better understand the additional factors that may contribute to phenotypic discordance. We are aware that the control groups are relatively small and the age among the control individuals varied, and we also acknowledge the fact that the DNAm profiles were obtained from blood DNA and that epigenetic marks can be cell type specific. We did our best to correct for cell type heterogeneity by using well-established bioinformatic tools. Finally, it is known that the cause of the VWS in the affected twins is the mutation reported by Kondo et al. and Jobling et al. (2011); however, this is the first study reporting a genome-wide epigenetic profiling of the syndrome, especially using the powerful discordant twin design.

Despite its limitations, the *in silico* functional analysis methods that we used, help us gain insight into the biology underlying the regions in which we detected differential methylation and allow us to explore whether pathways or processes are enriched among our best hits. We used over-representation analysis methods to identify potential pathways that could be affected by the observed changes in DNA methylation.

Clinical divergence between patients complicates diagnosis and genetic counseling, this is especially true when syndromic and nonsyndromic cases have overlapping clinical features, like VWS and nonsyndromic clefts. In addition to clinical implications and translational potential, studies of etiological factors of syndromic forms of clefting have greatly contributed to the understanding of the much more complex etiology of nonsyndromic cases. Therefore, our study emphasizes the need to understand the molecular mechanisms underlying phenotypic variability and the role of epigenetic factors in disease etiology.

## Data Availability

The original contributions presented in the study are included in the article/**Supplementary Material**, further inquiries can be directed to the corresponding author.
