# Supplementary material for: DNA methylation differences in monozygotic twins with Van der Woude syndrome
Source: Front Dent Med. 2023 Feb 17;4:1120948. doi: 10.3389/fdmed.2023.1120948 (PMC10019782; doi:10.3389/fdmed.2023.1120948)
Supplement: Supplementary file 1 [file Table1.docx]

**Supplementary Table S1.** List of genes previously associated with orofacial clefts based on human and mouse data.

| **Gene/locus** | **coordinates (hg19)** | **coordinates (hg38)** |
| --- | --- | --- |
| SKI | chr1:2159758-2241652 | chr1:2228319-2310213 |
| PRDM16 | chr1:2985732-3355185 | chr1:3069168-3438621 |
| MTHFR | chr1:11845780-11866512 | chr1:11785723-11806455 |
| PAX7 | chr1:18957340-19075360 | chr1:18630846-18748866 |
| CAPZB | chr1:19665269-19812033 | chr1:19338775-19485539 |
| KDM1A | chr1:23345936-23410182 | chr1:23019443-23083689 |
| GRHL3 | chr1:24526048-24690972 | chr1:24199558-24364482 |
| AHDC1 | chr1:27860546-27930942 | chr1:27534035-27604431 |
| COL9A2 | chr1:40766161-40783485 | chr1:40300489-40317813 |
| RIMS3 | chr1:41086352-41131354 | chr1:40620680-40665682 |
| WDR65 | chr1:43638001-43720029 | chr1:43172330-43254358 |
| LRP8 | chr1:53708036-53794141 | chr1:53242364-53328469 |
| DAB1 | chr1:57460451-59012406 | chr1:56994778-58546734 |
| PGM1 | chr1:64059082-64125916 | chr1:63593411-63660245 |
| SERBP1 | chr1:67873493-67896098 | chr1:67407810-67430415 |
| GNG12 | chr1:68167158-68299150 | chr1:67701475-67833467 |
| LHX8 | chr1:75594119-75627218 | chr1:75128434-75161533 |
| RPL5 | chr1:93297570-93307481 | chr1:92832013-92841924 |
| ABCA4 | chr1:94458390-94586704 | chr1:93992834-94121148 |
| ARHGAP29 | chr1:94614544-94740624 | chr1:94148988-94275068 |
| COL11A1 | chr1:103342023-103574428 | chr1:102876467-103108872 |
| CELSR2 | chr1:109792161-109818373 | chr1:109249539-109275751 |
| GNAI3 | chr1:110091237-110142817 | chr1:109548615-109600195 |
| ALX3 | chr1:110602492-110613294 | chr1:110059870-110070672 |
| PHGDH | chr1:120191034-120286841 | chr1:119648411-119744218 |
| NOTCH2 | chr1:120454176-120643366 | chr1:119911553-120100779 |
| SF3B4 | chr1:149895209-149899695 | chr1:149923317-149927803 |
| DENND4B | chr1:153901977-153919194 | chr1:153929501-153946718 |
| 1q23 | chr1:157061160-157468300 | chr1:157091368-157498510 |
| PBX1 | chr1:164524821-164868533 | chr1:164555584-164899296 |
| TMCO1 | chr1:165693530-165796992 | chr1:165724293-165827755 |
| DDX59 | chr1:200593024-200639035 | chr1:200623896-200669907 |
| REN | chr1:204123947-204159452 | chr1:204154819-204190324 |
| TRAF3IP3 | chr1:209929377-209955665 | chr1:209756032-209782320 |
| IRF6 | chr1:209958962-209979520 | chr1:209785617-209806175 |
| MCS9.7 | chr1:209989132-209989738 | chr1:209815787-209816393 |
| TGFB2 | chr1:218518678-218617961 | chr1:218345336-218444619 |
| COLEC11 | chr2:3642422-3692234 | chr2:3594832-3644644 |
| TAF1B | chr2:9983572-10074545 | chr2:9843443-9934416 |
| NBAS | chr2:15307040-15701464 | chr2:15166916-15561340 |
| FAM49A | chr2:16730727-16847102 | chr2:16549459-16665834 |
| WDR35 | chr2:20110024-20189866 | chr2:19910263-19990105 |
| CYP1B1 | chr2:38294116-38337044 | chr2:38066973-38109902 |
| ZFP36L2 | chr2:43449541-43453745 | chr2:43222402-43226606 |
| THADA | chr2:43457990-43823177 | chr2:43230851-43596038 |
| PREPL | chr2:44543420-44589001 | chr2:44316281-44361862 |
| SIX3 | chr2:45168841-45173210 | chr2:44941702-44946071 |
| PPP1R21 | chr2:48667737-48742530 | chr2:48440598-48515391 |
| TGFA | chr2:70674416-70781325 | chr2:70447284-70554193 |
| MRPL53 | chr2:74699109-74699814 | chr2:74471982-74472687 |
| LOXL3 | chr2:74759385-74782817 | chr2:74532258-74555690 |
| M1AP | chr2:74785010-74875465 | chr2:74557883-74648338 |
| CTNNA2 | chr2:79412357-80875986 | chr2:79185231-80648861 |
| GLI2 | chr2:121493199-121750229 | chr2:120735623-120992653 |
| HPE1 | chr2:128994290-129076442 | chr2:128236716-128318868 |
| HS6ST1 | chr2:128994290-129076442 | chr2:128236716-128318868 |
| ZEB2 | chr2:145121931-145278624 | chr2:144364364-144521057 |
| SCN2A | chr2:166051503-166248820 | chr2:165194993-165392310 |
| SCN1A | chr2:166841151-167005724 | chr2:165984641-166149214 |
| GAD1 | chr2:171669723-171717661 | chr2:170813213-170861151 |
| CHRNA1 | chr2:175612320-175652663 | chr2:174747592-174787935 |
| SATB2 | chr2:200134228-200335989 | chr2:199269505-199471266 |
| SUMO1 | chr2:203070905-203103322 | chr2:202206182-202238599 |
| ABCA12 | chr2:215796266-216003349 | chr2:214931542-215138626 |
| PAX3 | chr2:223064606-223163717 | chr2:222199887-222298998 |
| CHRNG | chr2:233404402-233412825 | chr2:232539692-232548115 |
| UGT1A9 | chr2:234580544-234681946 | chr2:233671898-233773300 |
| SNED1 | chr2:241938035-242034983 | chr2:240998618-241095568 |
| WNT7A | chr3:13857755-13921568 | chr3:13816258-13880071 |
| TGFBR2 | chr3:30648093-30735634 | chr3:30606601-30694142 |
| HYAL2 | chr3:50355221-50360213 | chr3:50317790-50322782 |
| WNT5A | chr3:55499743-55524567 | chr3:55465715-55490539 |
| FLNB | chr3:57994125-58157978 | chr3:58008398-58172251 |
| CADPS | chr3:62384021-62861091 | chr3:62398346-62875416 |
| EPHA3 | chr3:89156771-89531284 | chr3:89107621-89482134 |
| COL8A1 | chr3:99357319-99518070 | chr3:99638475-99799226 |
| FILIP1L | chr3:99547655-99833357 | chr3:99828811-100114513 |
| RYK | chr3:133784147-133969701 | chr3:134065303-134250859 |
| DVL3 | chr3:183873165-183891402 | chr3:184155377-184173614 |
| LIPH | chr3:185224050-185270376 | chr3:185506262-185552588 |
| MASP1 | chr3:186935070-187009768 | chr3:187217282-187291980 |
| TP63 | chr3:189349178-189615065 | chr3:189631389-189897276 |
| DLG1 | chr3:196769431-197026201 | chr3:197042560-197299330 |
| FGFR3 | chr4:1795020-1810599 | chr4:1793293-1808872 |
| RGS12 | chr4:3294748-3441640 | chr4:3293021-3439913 |
| MSX1 | chr4:4861392-4865663 | chr4:4859665-4863936 |
| EVC2 | chr4:5544499-5711275 | chr4:5542772-5709548 |
| PDGFRA | chr4:55095447-55164412 | chr4:54229280-54298245 |
| SHROOM3 | chr4:77356382-77704406 | chr4:76435229-76783253 |
| FRAS1 | chr4:78978477-79465423 | chr4:78057323-78544269 |
| ADH7 | chr4:100333418-100356667 | chr4:99412261-99435510 |
| PPP3CA | chr4:101944566-102269435 | chr4:101023409-101348278 |
| NUDT6 | chr4:123809852-123844123 | chr4:122888697-122922968 |
| SPRY1 | chr4:124317950-124324915 | chr4:123396795-123403760 |
| INTU | chr4:128544426-128647892 | chr4:127623271-127726737 |
| PDGFC | chr4:157681606-157892951 | chr4:156760454-156971799 |
| NEK1 | chr4:170290855-170533783 | chr4:169369704-169612632 |
| CLPTM1L | chr5:1317867-1345214 | chr5:1317752-1345099 |
| DNAH5 | chr5:13690437-14011927 | chr5:13690328-14011818 |
| 5p13.2 | chr5:34987678-36738268 | chr5:34987573-36738166 |
| UGT3A2 | chr5:36035123-36071460 | chr5:36035021-36071358 |
| NIPBL | chr5:36876871-37066515 | chr5:36876769-37066413 |
| CPLANE1 | chr5:37106330-37249478 | chr5:37106228-37249376 |
| FGF10 | chr5:44300349-44389808 | chr5:44300247-44389706 |
| ZSWIM6 | chr5:60628085-60841999 | chr5:61332258-61546172 |
| PIK3R1 | chr5:67511568-67597649 | chr5:68215740-68301821 |
| IQGAP2 | chr5:75699110-76003957 | chr5:76403285-76708132 |
| SCAMP1 | chr5:77656435-77776562 | chr5:78360611-78480739 |
| LHFPL2 | chr5:77781038-78065844 | chr5:78485215-78770021 |
| LHFPL2 | chr5:77781038-78065844 | chr5:78485215-78770021 |
| SEPTIN8 | chr5:132086511-132142933 | chr5:132750819-132807241 |
| SLC25A2 | chr5:140682202-140683616 | chr5:141302635-141304049 |
| SPRY4 | chr5:141689992-141706020 | chr5:142310427-142326455 |
| ARHGAP26 | chr5:142149942-142608576 | chr5:142770377-143229011 |
| LRS | chr5:145492597-145562213 | chr5:146113034-146182650 |
| TCOF1 | chr5:149737192-149779871 | chr5:150357629-150400308 |
| MSX2 | chr5:174151585-174157899 | chr5:174724582-174730896 |
| DUSP22 | chr6:291630-351355 | chr6:291630-351355 |
| FOXF2 | chr6:1389811-1395838 | chr6:1389576-1395603 |
| TFAP2A | chr6:10393419-10419892 | chr6:10393186-10419659 |
| MAS1L | chr6:29454474-29455733 | chr6:29486697-29487956 |
| 6p22 | chr6:29574888-30383816 | chr6:29607111-30416039 |
| TUBB | chr6:30685212-30693315 | chr6:30717435-30725538 |
| HLA-B | chr6:31321649-31334844 | chr6:31353872-31367067 |
| COL11A2 | chr6:33130458-33160276 | chr6:33162681-33192499 |
| POLR1C | chr6:43477440-43530156 | chr6:43509702-43562419 |
| ICK | chr6:52866101-52926622 | chr6:53001303-53061824 |
| BAI3 | chr6:69345174-70099398 | chr6:68635282-69389506 |
| COL9A1 | chr6:70925743-71012787 | chr6:70216040-70303084 |
| ZBTB24 | chr6:109781835-109804442 | chr6:109460632-109483239 |
| GJA1 | chr6:121756741-121770873 | chr6:121435595-121449727 |
| ESR1 | chr6:151977826-152450754 | chr6:151656691-152129619 |
| PARK2 | chr6:161768449-163148798 | chr6:161347417-162727766 |
| FAM20C | chr7:192969-300740 | chr7:192571-260772 |
| ACTB | chr7:5566040-5603533 | chr7:5526409-5563902 |
| AHR | chr7:16955983-17385776 | chr7:16916359-17346152 |
| SP8 | chr7:20821899-20826505 | chr7:20782279-20786886 |
| HOXA1 | chr7:27132612-27135619 | chr7:27092993-27096000 |
| HOXA2 | chr7:27139973-27142305 | chr7:27100354-27102686 |
| CHN2 | chr7:29186185-29553944 | chr7:29146569-29514328 |
| GLI3 | chr7:42000547-42303699 | chr7:41960949-42264100 |
| SEMA3E | chr7:82992554-83278455 | chr7:83363238-83649139 |
| ABCB1 | chr7:87132333-87342639 | chr7:87503017-87713323 |
| SEM1 | chr7:96110938-96339192 | chr7:96481626-96709880 |
| SHFM1 | chr7:96649708-96654143 | chr7:97020396-97024831 |
| COG5 | chr7:106841817-107204706 | chr7:107201372-107564261 |
| SHH | chr7:155592674-155605157 | chr7:155799980-155812463 |
| ESCO2 | chr8:27629466-27670157 | chr8:27771949-27812640 |
| NRG1 | chr8:31496738-32713184 | chr8:31639222-32855666 |
| FGFR1 | chr8:38257733-38326352 | chr8:38400215-38468834 |
| IKKα | chr8:42128820-42189978 | chr8:42271302-42332460 |
| SNTG1 | chr8:50822349-51709252 | chr8:49909789-50796692 |
| CHD7 | chr8:61591299-61780587 | chr8:60678740-60868028 |
| EYA1 | chr8:72109668-72504260 | chr8:71197433-71592025 |
| DCAF4L2 | chr8:88882975-88886243 | chr8:87870747-87874015 |
| MMP16 | chr8:89044239-89340254 | chr8:88032011-88328025 |
| RAD54B | chr8:95384188-95487343 | chr8:94371960-94475115 |
| VPS13B | chr8:100025494-100889808 | chr8:99013266-99877580 |
| FZD6 | chr8:104310661-104345094 | chr8:103298433-103332866 |
| 8q22.3 | chr8:104858878-105545709 | chr8:103846650-104533481 |
| RSPO2 | chr8:108911544-109095870 | chr8:107899316-108083642 |
| COLEC10 | chr8:120007691-120120694 | chr8:118995452-119108455 |
| MYC | chr8:128747680-128755197 | chr8:127735434-127742951 |
| 8q24 | chr8:128848399-131797730 | chr8:127836153-130785484 |
| LINC00976 | chr8:129965125-129978247 | chr8:128952879-128966001 |
| 9q21.32 | chr9:85800430-88446859 | chr9:83185515-85831944 |
| GAS1 | chr9:89559277-89562421 | chr9:86944362-86947506 |
| GADD45G | chr9:92219927-92221470 | chr9:89605012-89606555 |
| FAM120A | chr9:96213967-96328394 | chr9:93451685-93566112 |
| PTCH1 | chr9:98205262-98279339 | chr9:95442980-95517057 |
| FOXE1 | chr9:100615508-100618999 | chr9:97853226-97856717 |
| C9ORF156 | chr9:100659137-100684782 | chr9:97896855-97922500 |
| HEMGN | chr9:100689073-100707138 | chr9:97926791-97944856 |
| TGFBR1 | chr9:101866320-101916474 | chr9:99104038-99154192 |
| ABCA1 | chr9:107543287-107690436 | chr9:104781006-104928155 |
| FKTN | chr9:108320403-108416101 | chr9:105558122-105653820 |
| KLF4 | chr9:110247133-110252763 | chr9:107484852-107490482 |
| POMT1 | chr9:134378176-134399193 | chr9:131502789-131523806 |
| MYMK | chr9:136379708-136393734 | chr9:133514586-133528612 |
| PHYH | chr10:13319796-13344412 | chr10:13277796-13302412 |
| PTER | chr10:16478942-16555744 | chr10:16436943-16513745 |
| ACBD5 | chr10:27457064-27531975 | chr10:27168135-27243046 |
| PRKG1 | chr10:52750648-54058183 | chr10:50990888-52298423 |
| DKK1 | chr10:54074041-54077802 | chr10:52314281-52318042 |
| PCDH15 | chr10:55562531-57387702 | chr10:53802771-55627942 |
| ARID5B | chr10:63661443-63856703 | chr10:61901684-62096944 |
| BMPR1A | chr10:88516358-88692582 | chr10:86756601-86932825 |
| TCTN3 | chr10:97419580-97453900 | chr10:95659823-95694143 |
| CRTAC1 | chr10:99624757-99790585 | chr10:97865000-98030828 |
| FGF8 | chr10:103529866-103540128 | chr10:101770109-101780371 |
| SUFU | chr10:104263729-104393292 | chr10:102503972-102633535 |
| COL17A1 | chr10:105791044-105845638 | chr10:104031286-104085880 |
| KIAA1598 | chr10:118640988-118886097 | chr10:116881477-117126586 |
| VAX1 | chr10:118888032-118897812 | chr10:117128521-117138301 |
| WDR11 | chr10:122610817-122669036 | chr10:120851305-120909524 |
| FGFR2 | chr10:123237846-123357972 | chr10:121478332-121598458 |
| MGMT | chr10:131265454-131569247 | chr10:129467190-129770983 |
| DEAF1 | chr11:644233-707118 | chr11:644233-707118 |
| CDKN1C | chr11:2904443-2907005 | chr11:2883213-2885775 |
| PLEKHA7 | chr11:16798844-17035962 | chr11:16777297-17014415 |
| PAX6 | chr11:31806327-31839509 | chr11:31784779-31817961 |
| WT1 | chr11:32409321-32457110 | chr11:32387775-32435564 |
| CTNND1 | chr11:57520715-57587018 | chr11:57753243-57819546 |
| OVOL1 | chr11:65554534-65564685 | chr11:65787063-65797214 |
| TBX10 | chr11:67398774-67407234 | chr11:67631303-67639763 |
| DHCR7 | chr11:71139239-71163914 | chr11:71428193-71452868 |
| INPPL1 | chr11:71934745-71950191 | chr11:72223701-72239147 |
| C2CD3 | chr11:73723763-73882255 | chr11:74012718-74171210 |
| YAP1 | chr11:101981178-102104155 | chr11:102110447-102233424 |
| MMP3 | chr11:102706532-102714340 | chr11:102835801-102843609 |
| DYNC2H1 | chr11:102980139-103350591 | chr11:103109410-103479863 |
| KMT2A | chr11:118307171-118397547 | chr11:118436456-118526832 |
| NECTIN1 | chr11:119494120-119599910 | chr11:119623408-119729200 |
| PVRL1 | chr11:119508808-119599910 | chr11:119638098-119729200 |
| PKNOX2 | chr11:125034583-125303285 | chr11:125164687-125433389 |
| CDON | chr11:125825691-125933230 | chr11:125955796-126063335 |
| GLASS | chr11:134201768-134248235 | chr11:134331874-134378341 |
| GLB1L2 | chr11:134201768-134248235 | chr11:134331874-134378341 |
| LRP6 | chr12:12268959-12419978 | chr12:12116025-12267044 |
| PLEKHA5 | chr12:19282667-19529334 | chr12:19129733-19376400 |
| PTHLH | chr12:28111017-28125666 | chr12:27958084-27972733 |
| CNTN1 | chr12:41086241-41466217 | chr12:40692439-41072415 |
| PRICKLE1 | chr12:42850559-42984157 | chr12:42456757-42590355 |
| ADAMTS20 | chr12:43747669-43946006 | chr12:43353866-43552203 |
| COL2A1 | chr12:48366750-48398337 | chr12:47972967-48004554 |
| KMT2D | chr12:49412758-49454577 | chr12:49018975-49060794 |
| KRT8 | chr12:53290971-53343738 | chr12:52897187-52949954 |
| KRT18 | chr12:53342655-53346690 | chr12:52948871-52952906 |
| GDF11 | chr12:56136906-56151048 | chr12:55743122-55757264 |
| RPS26 | chr12:56435135-56438481 | chr12:56041351-56044697 |
| STAC3 | chr12:57637236-57644971 | chr12:57243453-57251188 |
| TMEM19 | chr12:72079862-72098827 | chr12:71686082-71705047 |
| ALX1 | chr12:85673998-85695562 | chr12:85280220-85301784 |
| DUSP6 | chr12:89741012-89746278 | chr12:89347235-89352501 |
| BTBD11 | chr12:107712198-108053419 | chr12:107318421-107659642 |
| ACACB | chr12:109554392-109706031 | chr12:109116587-109268226 |
| MMAB | chr12:109991520-110011385 | chr12:109553715-109573580 |
| MVK | chr12:110011060-110035930 | chr12:109573255-109598125 |
| TCTN2 | chr12:124155657-124192946 | chr12:123671110-123708399 |
| EP400 | chr12:132434487-132565005 | chr12:131949942-132080460 |
| IFT88 | chr13:21141277-21265583 | chr13:20567138-20691444 |
| POLR1D | chr13:28194879-28318374 | chr13:27620742-27744237 |
| B3GLCT | chr13:31774112-31906413 | chr13:31199975-31332276 |
| FREM2 | chr13:39261214-39461268 | chr13:38687077-38887131 |
| DACH1 | chr13:72012098-72441342 | chr13:71437966-71867204 |
| PIBF1 | chr13:73356271-73590599 | chr13:72782133-73016461 |
| KLF12 | chr13:74260226-74708066 | chr13:73686089-74133929 |
| LOC105370275 | chr13:80695926-80740529 | chr13:80121791-80166394 |
| SPRY2 | chr13:80910111-80915261 | chr13:80335976-80341126 |
| GPC6 | chr13:93879060-95060274 | chr13:93226807-94408020 |
| TGDS | chr13:95226308-95248496 | chr13:94574054-94596242 |
| 13q32.3 | chr13:100710303-101917007 | chr13:100058049-101264656 |
| ERCC5 | chr13:103498181-103528345 | chr13:102845831-102875995 |
| LINC00460 | chr13:107026825-107036663 | chr13:106374477-106384315 |
| PAX9 | chr14:37126773-37148567 | chr14:36657568-36679362 |
| ABHD12B | chr14:51338771-51371688 | chr14:50872053-50904970 |
| LINC00640 | chr14:51799786-51832275 | chr14:51333068-51365557 |
| BMP4 | chr14:54416454-54425479 | chr14:53949736-53958761 |
| KIAA0586 | chr14:58894103-59018015 | chr14:58427385-58551297 |
| SIX1 | chr14:61110139-61124977 | chr14:60643421-60658259 |
| TGFB3 | chr14:76424440-76449354 | chr14:75958097-75983011 |
| POMT2 | chr14:77741299-77787226 | chr14:77274956-77320883 |
| GSC | chr14:95234560-95236450 | chr14:94768223-94770113 |
| DICER1 | chr14:95552565-95624347 | chr14:95086228-95158010 |
| JAG2 | chr14:105607319-105635161 | chr14:105140982-105168824 |
| GABRB3 | chr15:26788693-27184686 | chr15:26543546-26939539 |
| CHRNA7 | chr15:32215641-32465219 | chr15:31923438-32173018 |
| GREM1 | chr15:33010205-33037307 | chr15:32718004-32745106 |
| ACTC1 | chr15:35082431-35087750 | chr15:34790230-34795549 |
| MEIS2 | chr15:37181405-37393500 | chr15:36889204-37101299 |
| SEMA6D | chr15:47476298-48066425 | chr15:47184101-47774228 |
| TPM1 | chr15:63334831-63364114 | chr15:63042632-63071915 |
| SMAD3 | chr15:67356101-67487511 | chr15:67063763-67195173 |
| ARID3B | chr15:74833547-74890472 | chr15:74541206-74598131 |
| IGF1R | chr15:99191768-99507759 | chr15:98648539-98964530 |
| UNKL | chr16:1413206-1464752 | chr16:1363205-1414751 |
| IFT140 | chr16:1560428-1662073 | chr16:1510427-1612072 |
| CREBBP | chr16:3775055-3930714 | chr16:3725054-3880713 |
| ADCY9 | chr16:4003388-4166443 | chr16:3953387-4116442 |
| SRCAP | chr16:30709530-30752730 | chr16:30698209-30741409 |
| RPGRIP1L | chr16:53632065-53737850 | chr16:53598153-53703938 |
| MMP2 | chr16:55423612-55540603 | chr16:55389700-55506691 |
| CBFB | chr16:67062887-67134961 | chr16:67028984-67101058 |
| ESRP2 | chr16:68262936-68272005 | chr16:68229033-68238102 |
| CDH1 | chr16:68771195-68869440 | chr16:68737292-68835537 |
| DTD | chr16:69743304-69760463 | chr16:69709401-69726560 |
| DHODH | chr16:72042487-72061563 | chr16:72008588-72027664 |
| BCAR1 | chr16:75262079-75301951 | chr16:75228181-75268053 |
| CFDP1 | chr16:75327596-75467401 | chr16:75293698-75433503 |
| MAF | chr16:79619740-79634634 | chr16:79585843-79600737 |
| CRISPLD2 | chr16:84853591-84954374 | chr16:84819985-84920768 |
| FOXC2 | chr16:86600435-86603334 | chr16:86566829-86569728 |
| KBGS | chr16:89334029-89556969 | chr16:89267621-89490561 |
| RBFOX3 | chr17:32402-56969 | chr17:79089345-79482598 |
| YWHAE | chr17:1247569-1303516 | chr17:1344275-1400222 |
| PIK3R5 | chr17:8782228-8869029 | chr17:8878911-8965712 |
| NTN1 | chr17:8924827-9147317 | chr17:9021510-9244000 |
| MYH3 | chr17:10531843-10560626 | chr17:10628526-10657309 |
| MEOX1 | chr17:41717757-41739290 | chr17:43640389-43661922 |
| FZD2 | chr17:42634852-42638630 | chr17:44557484-44561262 |
| EFTUD2 | chr17:42927316-42976813 | chr17:44849948-44899445 |
| KANSL1 | chr17:44107282-44302755 | chr17:46029916-46225389 |
| LRRC37A2 | chr17:44589834-44633012 | chr17:46512468-46555646 |
| WNT3 | chr17:44839872-44910520 | chr17:46762506-46833154 |
| WNT9B | chr17:44910567-44964096 | chr17:46833201-46886730 |
| GOSR2 | chr17:45000441-45052890 | chr17:46923075-46975524 |
| SCAP1 | chr17:46210804-46522423 | chr17:48133442-48445061 |
| DLX4 | chr17:48046334-48052323 | chr17:49968970-49974959 |
| NOG | chr17:54671060-54672972 | chr17:56593699-56595611 |
| TANC2 | chr17:61043596-61505064 | chr17:62966235-63427703 |
| SOX9 | chr17:70117161-70122557 | chr17:72121020-72126416 |
| SEPT9 | chr17:75277581-75496678 | chr17:77281499-77500596 |
| TIMP2 | chr17:76849059-76921469 | chr17:78852977-78925387 |
| EIF4A3 | chr17:78108168-78120950 | chr17:80134369-80147151 |
| ZNF750 | chr17:80787310-80797898 | chr17:82829434-82840022 |
| TYMS | chr18:657653-673578 | chr18:657653-673578 |
| SMCHD1 | chr18:2655725-2805015 | chr18:2655726-2805017 |
| TGIF1 | chr18:3411606-3459976 | chr18:3411608-3459978 |
| PIEZO2 | chr18:10666480-11149568 | chr18:10666483-11149569 |
| FAM38B | chr18:10670244-11149568 | chr18:10670247-11149569 |
| MIB1 | chr18:19284918-19450914 | chr18:21704957-21870953 |
| DSC3 | chr18:28569331-28622781 | chr18:30989365-31042815 |
| MAPRE2 | chr18:32556892-32723434 | chr18:34976928-35143470 |
| EPG5 | chr18:43380546-43547295 | chr18:45800581-45967329 |
| SMAD2 | chr18:45335328-45457517 | chr18:47808957-47931146 |
| NEDD4L | chr18:55711458-56068772 | chr18:58044226-58401540 |
| CDH19 | chr18:64168320-64271375 | chr18:66501083-66604138 |
| TXNL4A | chr18:77730813-77793949 | chr18:79970813-80033949 |
| STK11 | chr19:1177557-1228430 | chr19:1177558-1228431 |
| PTPRS | chr19:5158506-5340823 | chr19:5158495-5340812 |
| ZNF431 | chr19:21324827-21378856 | chr19:21142024-21196053 |
| CCNE1 | chr19:30302898-30315219 | chr19:29811991-29824312 |
| RHPN2 | chr19:33469498-33555794 | chr19:32978592-33064888 |
| RYR1 | chr19:38924331-39085913 | chr19:38433691-38595273 |
| LRFN1 | chr19:39797206-39811503 | chr19:39306566-39320863 |
| TGFB1 | chr19:41807492-41859827 | chr19:41301587-41353922 |
| PVR | chr19:45147098-45169430 | chr19:44643798-44666162 |
| BCL3 | chr19:45250962-45263301 | chr19:44747705-44760044 |
| NECTIN2 | chr19:45349432-45392480 | chr19:44846175-44889223 |
| CLPTM1 | chr19:45457842-45496599 | chr19:44954585-44993341 |
| SIX5 | chr19:46268043-46272510 | chr19:45764785-45769252 |
| FKRP | chr19:47249303-47280245 | chr19:46746046-46776988 |
| SULT2A1 | chr19:48373724-48389572 | chr19:47870467-47886315 |
| GP6 | chr19:55525073-55549632 | chr19:55013705-55038264 |
| AVP | chr20:3063202-3065370 | chr20:3082556-3084724 |
| BMP2 | chr20:6748333-6760893 | chr20:6767686-6780246 |
| PLCB4 | chr20:9048472-9485240 | chr20:9067825-9504593 |
| ISM1 | chr20:13201921-13281298 | chr20:13221274-13300651 |
| DEFB124 | chr20:30053274-30064560 | chr20:31465471-31476757 |
| ASXL1 | chr20:30946133-31027122 | chr20:32358330-32439319 |
| ACSS2 | chr20:33459949-33515765 | chr20:34872146-34927962 |
| DHX35 | chr20:37590942-37668366 | chr20:38962299-39039723 |
| MAFB | chr20:39314488-39317876 | chr20:40685848-40689236 |
| PMEPA1 | chr20:56223448-56286592 | chr20:57648392-57711536 |
| COL9A3 | chr20:61447596-61472511 | chr20:62816244-62841159 |
| RIPK4 | chr21:43159529-43187249 | chr21:41739369-41767089 |
| PDE9A | chr21:44073731-44195619 | chr21:42653621-42775509 |
| SLC19A1 | chr21:46913486-46993279 | chr21:45493572-45573365 |
| COL6A2 | chr21:47518026-47552762 | chr21:46098112-46132848 |
| UFD1 | chr22:19437434-19466725 | chr22:19449911-19479202 |
| TBX1 | chr22:19744226-19771116 | chr22:19756703-19783593 |
| TXNRD2 | chr22:19863040-19929343 | chr22:19875517-19941820 |
| SNAP29 | chr22:21213295-21245502 | chr22:20859007-20891214 |
| SPECC1L | chr22:24666785-24813707 | chr22:24270817-24417739 |
| MN1 | chr22:28144265-28197744 | chr22:27748277-27801756 |
| MYH9 | chr22:36677326-36784055 | chr22:36281280-36388010 |
| FBLN1 | chr22:45898118-45997015 | chr22:45502238-45601135 |
| ANOS1 | chrX:8496915-8700178 | chrX:8528874-8732137 |
| KAL1 | chrX:8496915-8700178 | chrX:8528874-8732137 |
| MID1 | chrX:10413350-10851802 | chrX:10445310-10833683 |
| AMELX | chrX:11311533-11327708 | chrX:11293413-11309588 |
| OFD1 | chrX:13732624-13791857 | chrX:13714505-13773738 |
| PIGA | chrX:15337573-15353676 | chrX:15319451-15335554 |
| MBTPS2 | chrX:21857735-21903541 | chrX:21839617-21885423 |
| BCOR | chrX:39910499-40036582 | chrX:40051246-40177329 |
| KDM6A | chrX:44732434-44972024 | chrX:44873188-45112779 |
| PORCN | chrX:48367380-48379202 | chrX:48508992-48520814 |
| SMC1A | chrX:53401070-53449677 | chrX:53374149-53422728 |
| PHF8 | chrX:53963113-54075369 | chrX:53936680-54048936 |
| FGD1 | chrX:54471887-54522667 | chrX:54445454-54496234 |
| AMER1 | chrX:63404997-63425588 | chrX:64185117-64205708 |
| HDAC8 | chrX:71549366-71792693 | chrX:72329516-72572843 |
| TBX22 | chrX:79270252-79287273 | chrX:80014753-80031774 |
| STAG2 | chrX:123094410-123236506 | chrX:123960560-124102656 |
| GPC3 | chrX:132669773-133119621 | chrX:133535745-133985594 |
| FLNA | chrX:153576899-153603002 | chrX:154348531-154374634 |
| FMD | chrX:153576899-153603002 | chrX:154348531-154374634 |
| OPD1 | chrX:153576899-153603002 | chrX:154348531-154374634 |
